# Calcification traits for cryptic species identification: Insights into coralline biomineralization

**DOI:** 10.1371/journal.pone.0273505

**Published:** 2022-10-03

**Authors:** Daniela Basso, Giulia Piazza, Valentina Alice Bracchi

**Affiliations:** 1 Department of Earth and Environmental Sciences, University of Milano-Bicocca, Milan, Italy; 2 CoNISMa Research Unit of Milano-Bicocca, Milano, Italy; 3 Department of Earth and Ocean Dynamics, University of Barcelona, Barcelona, Spain; CIIMAR Interdisciplinary Centre of Marine and Environmental Research of the University of Porto, PORTUGAL

## Abstract

Calcareous red algae are foundation species and ecosystem engineers with a global distribution. The principles governing their calcification pathways are still debated and the morphological characters are frequently unreliable for species segregation, as shown by molecular genetics. The recent description of the new species *Lithophyllum pseudoracemus*, previously undetected and morphologically confused with *Lithophyllum racemus*, offered a challenging opportunity to test the effectiveness of microanatomy and ultrastructural calcification traits as tools for the identification of these two species, for integrative taxonomy. High resolution SEM images of molecularly identified samples showed that the different size of the perithallial cells and the features of the asexual conceptacle chambers may contribute to the separation of the two species. The two species share the same crystallite morphology in the primary and secondary cell-wall calcification, as previously described in other species belonging to the same clade. However, the perithallial secondary calcification was significantly thicker in *L*. *racemus* than in *L*. *pseudoracemus*. We described a granular calcified layer in the innermost part of the cell wall, as a putative precursor phase in the biomineralization and formation of the secondary calcification. The hypothesis of different pathways for the formation of the primary and secondary calcification is supported by the observed cell elongation associated with thicker and higher Mg/Ca primary calcification, the inverse correlation of primary and secondary calcification thickness, and the absence of primary calcification in the newly formed wall cutting off an epithallial cell from the meristem.

## Introduction

Non-geniculate “crustose” coralline algae (CCA) have a widely recognized global ecological importance and provide multiple ecosystem services, which include, among others, their role as habitat formers [1–8] and carbonate producers [9–14]. These properties derive from CCA ability to precipitate high-magnesium calcite (containing more than 8–12 mol% MgCO_3_) in the cell walls, along polysaccharide microfibrils [12, 15–18]. The mineralized thallus offers strength and protection to the alga against grazing and other disturbances [19] and can be preserved in the fossil record through geological times [14, 20–24]. During biomineralization, CCA incorporate several major and trace elements that mirror the chemical properties of their growth environment, becoming significant proxy records for paleoecology and palaeoceanography [25–28]. Longitudinal sections of some CCA species show dark and light bands (= thallus zonation), especially obvious in long protuberances, reflecting a rhythmic, seasonal growth pattern [1, 29–32]. Dark bands are formed by thick-walled small cells, while light bands are made of longer, thin-walled cells. Annual growth bands can be used for establishing the age of the CCA and for high-resolution time series analyses [25, 33, 34]. Cell wall calcification is composed of radial high-Mg calcite crystallites, around the cell lumen, and other tangentially arranged crystallites, the latter typically visible at the boundary between two adjacent cell filaments [18, 35–39].

CCA species identification merely based on morphological descriptors can lead to uncertainties, as highlighted by several systematic revisions based on molecular genetics [40–47]. In other calcifying marine organisms such as foraminifera [48–50], coccolithophores [51] and corals [52–55], the pattern of mineralization has been used for taxonomic identification, showing consistency with molecular phylogeny. Recently, Auer and Piller [38] matched the micro- and nanomorphology of CCA cell walls with molecular phylogeny, grouping different genera based on the crystallite shape in the epithallial and meristematic cell walls. Moreover, recent studies on the cell wall ultrastructure of *Lithothamnion corallioides* (P. Crouan & H. Crouan) P. Crouan & H. Crouan revealed a consistent pattern in the crystallite shape and arrangement, apparently unaffected by the environment [39].

CCA are common in Mediterranean benthic communities, where they are distributed from the intertidal zone down to circalittoral depths [56, 57]. Nevertheless, their calcification process is still poorly studied. *Lithophyllum racemus* (Lamarck) Foslie is a Mediterranean endemic non-geniculate CCA, forming unattached globular rhodoliths, showing a typical “praline” morphotype [56, 58–64]. A phylogenetic study [65] revealed the cryptic species *Lithophyllum pseudoracemus* Caragnano, Rodondi & Rindi, that had been previously confounded with the morphologically similar *L*. *racemus*. The two morphologically similar but genetically distinct species *L*. *racemus* and *L*. *pseudoracemus* may occur sympatrically and provide an opportunity to test the suitability of putative descriptors relying on calcification traits as a new taxonomic character for CCA identification at species level. Beside the ultrastructural calcification traits, we considered and measured different classical morphological descriptors that may help in species identification., such as cell and conceptacle microanatomy. Both molecularly classified samples of *L*. *racemus* and *L*. *pseudoracemus* [65], and other morphoanatomically similar samples that lack phylogenetic characterization were investigated.

## Materials and methods

### Sample collection

Samples were collected at different locations across the Western and Eastern Mediterranean Sea (Table 1). They were recovered in the framework of the Italian “Marine Strategy” Programme and by local surveys. The collection of rhodoliths of *Lithophyllum racemus* and *L*. *pseudoracemus* does not require a permit. Four out of six samples have been targeted for a multi-gene molecular phylogeny at Università Politecnica delle Marche (AN) [65], and identified as *L*. *racemus* and *L*. *pseudoracemus* (Table 1). Sample DB661 is the neotype of *L*. *racemus* [59]. The samples used for phylogenetic analyses are currently stored in the Herbarium Universitatis Florentinae, at the Natural History Museum in Florence (Italy). The last two samples, referred to as *L*. cf. *racemus* (DB865) and *L*. cf. *pseudoracemus* (DB866), were not molecularly classified (Table 1).

**Table 1:** *L*. *racemus*, *L*. *pseudoracemus*, *L*. cf. *pseudoracemus* and *L*. cf. *racemus* specimens examined under SEM.

Sample	Herbarium	Sampling site (latitude, longitude)	Depth (m)	Species
DB661	FI058887	Capri, Gulf of Naples (Italy) (40°34’08”N, 14°13’32”E)	50	*Lithophyllum racemus*
DB867	FI058894	Pontian Isl. (Italy) (40°54’47”N, 12°52’58”E)	64.9	*Lithophyllum racemus*
DB835	FI058891	Villasimius, Sardinia (Italy) (39°08′32″N, 9°31′14″E)	40	*Lithophyllum pseudoracemus*
DB768	FI058890	Pontian Isl. (Italy) (40°11’43”N, 12°53’07”E)	66.4	*Lithophyllum pseudoracemus*
DB865	-	Sveta Katarina, Rovinj (Croatia) (45°04’32”N, 13°37’38”E)	10	*Lithophyllum* cf. *racemus*
DB866	-	Torre dell’Orso, Puglia (Italy) (40°14’00”N, 18°28’00”E)	44	*Lithophyllum* cf. *pseudoracemus*

### SEM image analysis

Specimens were washed and air-dried and then prepared for Scanning Electron Microscopy (SEM) according to [39]. The samples were fragmented along the growth direction, mounted on stubs by mean of graphite paste and finally chrome-coated before analysis under a Field Emission Gun SEM (SEM-FEG) Gemini 500 Zeiss at the University of Milano-Bicocca.

**Fig 1:**
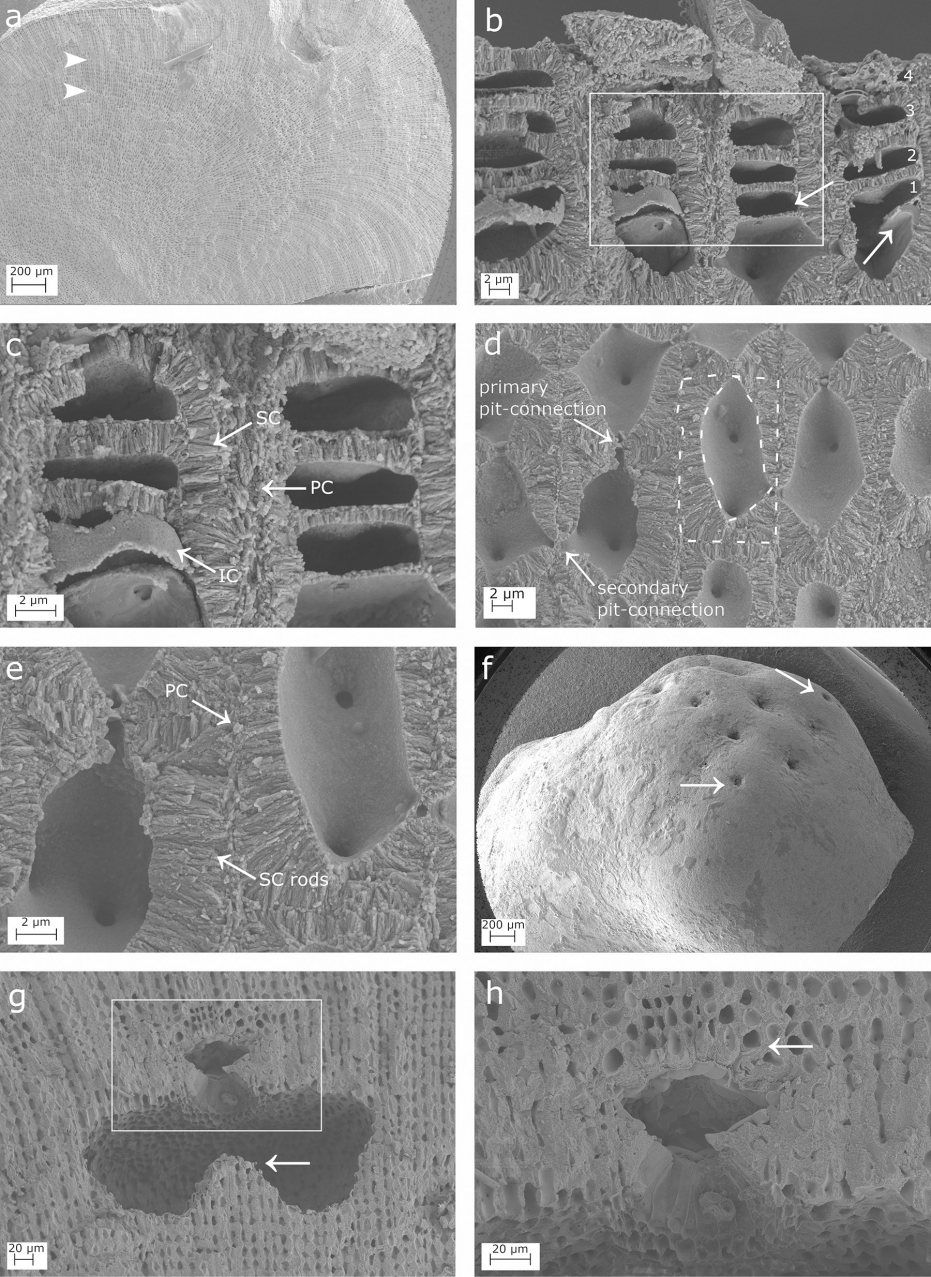
SEM images showing longitudinal sections of *Lithophyllum racemus*. (a) Fountain pattern in the arrangement of perithallial cells; two arrowheads indicate a growth band; (b) Four superimposed epithallial cells (numbered). Note the evidence of meristematic division (white arrows); (c) magnification of the inset in (b) to show: a primary (PC), secondary (SC) and inner calcification (IC); (d) perithallial cells with primary and secondary pit-connections (arrows); the cell wall area corresponds to the surface included between the two dashed lines; (e) magnification of (d) to show perithallial thin PC and rod-shaped crystallites of the SC; (f) conceptacles weakly protruding from thallus surface (white arrows); (g) a conceptacle chamber showing a well-developed calcified columella (arrow); (h) magnification of the inset in (g) showing the secondary hypothallus (arrow).

Hereinafter, short cells will be referred to as slow-growing cells constituting dark bands and produced in the cold season; long cells, instead, will be referred to as fast-growing cells constituting light bands and produced in the warm season [1, 30, 33, 37, 39]. Short cells are generally described as smaller, with thicker calcification and with lower Mg/Ca ratio, compared to long cells [33, 37, 39]. Information about biometry and calcification was extracted from 370 SEM images from longitudinal or transverse sections of protuberances. In the descriptions, L denotes the length, H is the height, D is the diameter. Measurements included the size of epithallial and perithallial cell [66] and cell lumen [39], the conceptacle size [67], the pore canal length, the number of cells in the pore canal filament and the number of epithallial cells (Table 2). Other classical descriptors such as the cell, conceptacle and pore canal shapes, pit-connections, conceptacle elevation, and presence and development of a calcified columella were also described. For the description of the columella development, we indicate as H the conceptacle height according to Adey and Adey [67], while the distance between the top of the columella and the conceptacle roof is indicated as h1, as in Basso et al. [59]. Within the calcified cell wall, primary calcification (PC) refers to the outer layer of Mg-calcite, formed by crystallites arranged parallel to the cell membrane (Fig 1) (= PW in [39]). Secondary calcification (SC) refers, instead, to the calcified layer which accounts for most of the cell wall thickness and is constituted of crystallites radial to the cell membrane and normal to the PC crystallites (Fig 1C; SW in [39]). In SEM images of longitudinal sections of algal protuberances, the cell wall area is measured as the calcified surface area surrounding the cell lumen and delimited by the outer borders of the cell (Fig 1D). The boundaries of each cell run at the half of the PC, which may be very thin (Fig 1E). The calcification was analysed mainly in epithallial and perithallial cells. The occurrence of thallus growth over an old conceptacle gave the opportunity to observe the cells of the secondary hypothallus [68], where the PC was particularly evident. The measured calcification traits included the SC maximum thickness, the PC maximum thickness and the cell wall area of epithallial and perithallial cells. Moreover, the shape and size of primary crystallites (= those forming the PC), the shape of secondary crystallites (= forming the SC) were also described. The same information was extracted for short and long cells separately in specimen DB867.

**Table 2:** Depth of sample collection and mean values (±SD) of biometrical traits from SEM image (μm).

	*Lithophyllum racemus*		*Lithophyllum pseudoracemus*		*L*. cf. *pseudoracemus*	*L*. cf. *racemus*
	DB661	DB867	DB835	DB768	DB866	DB865
**Depth (m)**	50	64.9	40	66.4	44	10
Perithallial cell (LxD)	17.9 ± 4.3 x 11.2 ± 2.4 (N = 39)	27.2 ± 4.6 x 13.2 ± 3.3 (N = 15)	15.4 ± 1.6 x 10.5 ± 1.6 (N = 42)	16.28 ± 2.8 x 10.79 ± 1.2 (N = 22)	21.1 ± 2.5 x 11.8 ± 1.8 (N = 34)	20.5 ± 2.5 x 2.5 ± 2.5 (N = 19)
Perithallial cell lumen (LxD)	13.8 ± 3.2 x 6.3 ± 1.2 (N = 51)	22.1 ± 3.7 x 7.1 ± 1.9 (N = 32)	12.5 ± 2.0 x 4.9 ± 0.9 (N = 63)	14.06 ± 2.6 x 5.43 ± 1.1 (N = 58)	19.4 ± 2.8 x 7.3 ± 1.1 (N = 34)	19.8 ± 2.5 x 7.6 ± 1.2 (N = 23)
Tetra/bisporangial conceptacle chamber (DxH)	275.3 ± 35.5 x 112.5 ± 19.3 (N = 23)	321.9 x 120.8 (N = 1)	261.1 ± 5.9 x 123.7 ± 22.7 (N = 3)	-	318.9 ± 20.3 x 142.0 ± 11.4 (N = 3)	325.1 x 136.9 (N = 1)
Pore canal length	40.9 ± 3.0 (N = 6)	-	41.7 (N = 1)	-	46.8 ± 6.4 (N = 3)	37.5 (N = 1)
Pore canal cells (n)	3–4	-	3	-	2–3	3
Epithallial cell (LxD)	3.7 ± 0.9 x 11.5 ± 0.7 (N = 5)	2.9 ± 0.6 x 12.6 ± 0.6 (N = 3)	3.8 ± 0.5 x 10.1 ± 1.0 (N = 3)	5.21 ± 0.4 x 9.03 ± 0.7 (N = 3)	5.2 x 11.3 (N = 1)	4.8 ± 1.0 x 13.1 ± 1.6 (N = 8)
Epithallial cell lumen (LxD)	2.1 ± 0.4 x 7.5 ± 1.4 (N = 18)	1.3 ± 0.1 x 8.9 ± 0.1 (N = 4)	2.3 ± 0.4 x 7.2 ± 1.1 (N = 7)	2.75 ± 0.5 x 6.16 ± 0.6 (N = 5)	3.0 ± 0.6 x 8.6 ± 2.8 (N = 5)	2.3 ± 0.9 x 8.6 ± 1.4 (N = 9)
Epithallial cells (n)	3, up to 5	3	1 or 2	1 or 2	1 or 2	1 or 2

### Statistical analysis

Principal Component Analysis (PCA) was performed on differently combined datasets. A first dataset included only biometrical data; the second only data on calcification traits and the last dataset included both biometrical and calcification data. Biometrical data comprised epithallial and perithallial cell size. Calcification data, instead, included the SC thickness and the cell wall area in both epithallial and perithallial cells, as well as the PC thickness. The PCA analysis was run in MATLAB R2020a software using the PCA toolbox released by [69]. Moreover, parametric and non-parametric univariate analysis of variance was applied to both biometrical and calcification dataset, in order to evaluate the statistical significance of the differences among group medians and means. Univariate analysis was also applied to compare results from short and long cells. In particular, the Kruskal-Wallis test followed by the Dunn’s test for comparisons and the One-way ANOVA followed by the Tukey’s test for post-hoc analysis, were performed in R 3.6.3 software.

## Results

### SEM image analyses

Scanning electron microscope (SEM) imaging of the cell walls showed similar shape of calcite crystallites within *L*. *racemus* and *L*. *pseudoracemus* thalli. A detailed description of each sample, including observations on both biometrical parameters and calcification traits, is provided below.

***Lithophyllum racemus*.** Microscopic anatomy: specimens were pink in colour, non-geniculate and fruticose with protuberances up to 1 cm long (up to 5 mm in DB867). A longitudinal section of protuberances showed perithallial filaments with a “fountain” pattern, with rows of cells which ran parallel and then diverge towards the thallus surface following opposite directions (Fig 1A). Dark and light growth bands were also detected (Fig 1A; [65]). Three superposed flattened epithallial cells were frequently observed (up to 5 in DB661; Fig 1B and 1C), L 2.92–3.72 μm and D 11.48–12.59 μm (Table 2). Perithallial cells were connected by primary and secondary pit-connections and lacked cell fusions (Fig 1D and 1E). Perithallial cells were rectangular in longitudinal section, L 17.87–27.20 μm and D 11.16–13.23 μm (Fig 1D and 1E; Table 2). Trichocytes were not observed. Many asexual uniporate conceptacles weakly protruded from the surface, with a sunken pore aperture (Fig 1F). The conceptacle chamber was kidney-shaped in longitudinal medial section, D 275.34–321.93 μm and H 112.48–120.82 μm (Fig 1G; Table 2), giving a mean D/H ratio of 2.55. A well-defined calcified columella rose from the conceptacle floor (Fig 1G). The columella had a height exceeding half of the height of the conceptacle chamber (H), therefore the distance between the columella top and the chamber roof (h1 in [59] was < H/2. The pore canal was medially cut and observed only in the neotype DB661. It was conical, appearing triangular in section, about 41 μm long, bordered by filaments composed of 3–4 cells, and showing a rhomboidal enlarged cavity on its top (Fig 1H; Table 2; [70]).

Calcification: the epithallial cells (Fig 1B) were strongly calcified with a cell wall area of 30.17–174.39 μm^2^ (Table 3). The PC between different cell filaments was composed of rod-shaped crystallites running more or less parallel to the cell membrane (Fig 1C), while the PC between epithallial cells of the same filament was very thin and hardly visible. The SC consisted of one layer of rods perpendicular to the cell membrane, locally appearing as flattened, loosely packed elements (Fig 1C). They determined an SC maximum thickness of 1.54 ± 0.3 μm (Table 3). We observed an additional, distinct, innermost thin calcified layer (= IC) in contact with the plasma membrane, composed of irregular granules with crenate margins and appearing as a granular surface bordering the cell lumen (Fig 1C). The meristematic cell, showing primary and secondary pit-connections, is superposed by several epithallial cells connected by exclusively primary pit-connections (Fig 1B). The meristem cell and its several generations of superposed epithallial cells shared the same PC and thick SC, as observed at the boundary with the adjacent cell-filament. The cut-off of a new epithallial cell occurs by transversal division of the upper (distal) part of the meristematic cell. The weak calcification of the newly formed horizontal portion of the cell membrane was formed exclusively by the IC (Fig 1B and 1C), with a later formation and then progressive thickening of the SC in older epithallial cells (Fig 1B and 1C).

**Table 3:** Mean values (±SD) of calcification traits from SEM images (μm, otherwise differently specified).

		*Lithophyllum racemus*		*Lithophyllum pseudoracemus*		*L*. cf. *pseudoracemus*	*L*. cf. *racemus*
		DB661	DB867	DB835	DB768	DB866	DB865
**Depth (m)**		50	64.9	40	66.4	44	10
Perithallial cells	SC thickness	2.7 ± 0.6 (N = 50)	2.9 ± 0.6 (N = 31)	2.5 ± 0.4 (N = 62)	2.3 ± 0.4 (N = 57)	2.4 ± 0.5 (N = 34)	2.9 ± 0.6 (N = 23)
	PC thickness	0.5 ± 0.2 (N = 28)	0.9 ± 0.2 (N = 35)	1.0 ± 0.3 (N = 31)	1.6 ± 0.5 (N = 54)	1.2 ± 0.3 (N = 23)	1.3 ± 0.3 (N = 24)
	Cell wall area (μm^2^)	116.6 ± 34.7 (N = 39)	174.4 ± 50.2 (N = 24)	99.7 ± 18.9 (N = 35)	100.5 ± 21.3 (N = 23)	117.7 ± 26.0 (N = 22)	137.8 ± 29.2 (N = 21)
Epithallial cells	SC thickness	1.5 ± 0.3 (N = 18)	1.4 ± 0.2 (N = 3)	1.0 ± 0.3 (N = 3)	0.9 ± 0.2 (N = 5)	1.0 ± 0.4 (N = 4)	1.1 ± 0.2 (N = 9)
	Cell wall area (μm^2^)	30.2 ± 8.4 (N = 6)	26.0 ± 8.2 (N = 3)	25.0 ± 9.7 (N = 5)	25.0 ± 8.3 (N = 6)	27.2 ± 8.3 (N = 4)	35.8 ± 12.0 (N = 7)

**Fig 2:**
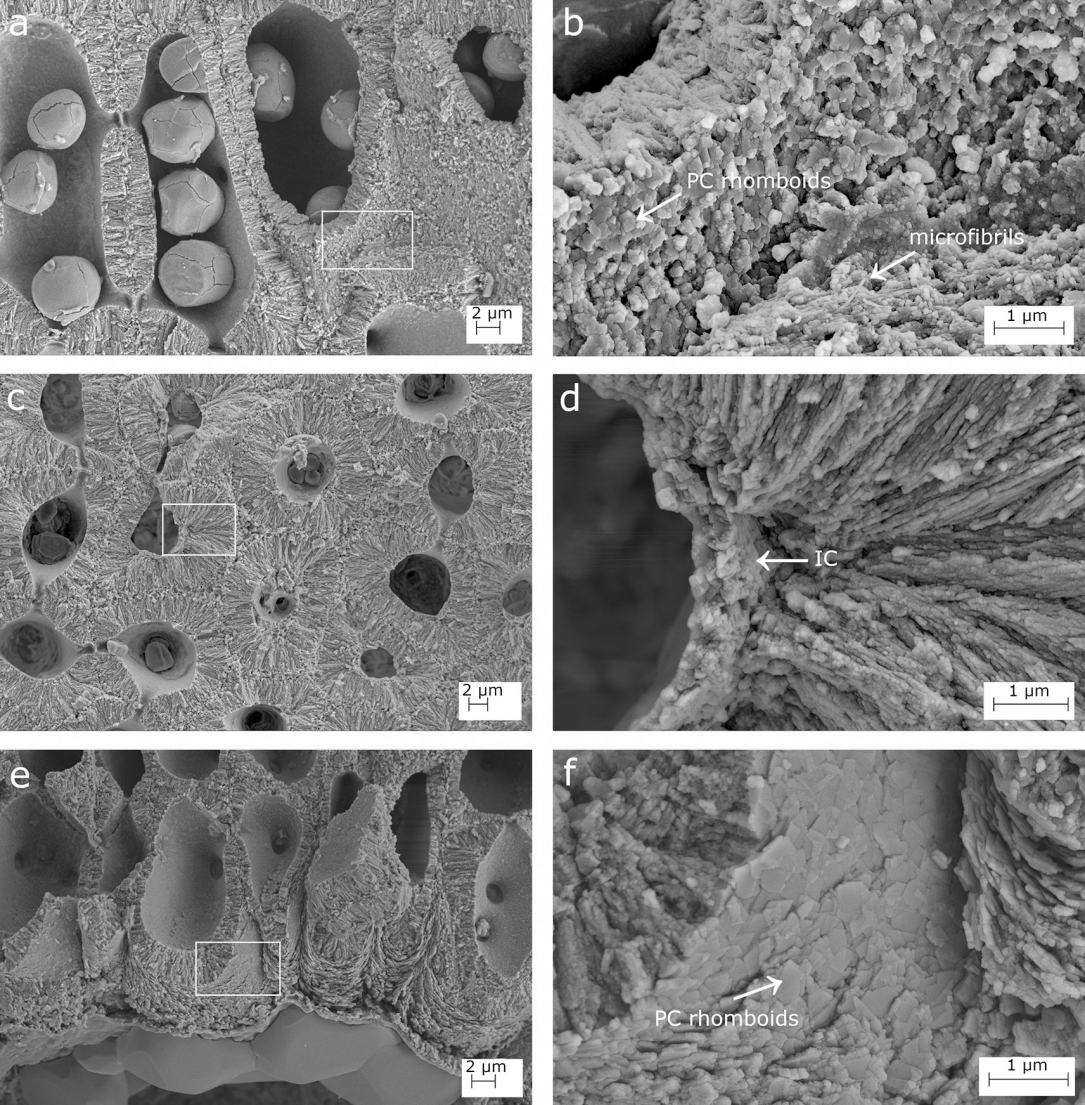
SEM details of the calcification in *Lithophyllum racemus*. (a) Calcified perithallial cells; (b) magnification of the inset in (a): rhomboidal crystallites of the primary calcification (PC, arrow) embedded in organic microfibrils (arrow); (c) transverse section of the perithallus; (d) magnification of the inset in (c) showing the innermost calcified layer (IC, arrow); (e) secondary hypothallus over the conceptacle pore canal in Fig 1H; (f) magnification of the inset in (e): regular rhomboidal crystallites of the hypothallial PC (arrow).

The perithallial cells in longitudinal section had oblong lumen and strongly calcified cell walls, with an area of 116.57–174.39 μm^2^ (Fig 1D and 1E; Table 3). The perithallial PC is composed of packed irregular rhomboids with smooth indented margins, oriented parallel to the cell membrane (Fig 2A and 2B). The PC measured between two adjacent cell filaments was 0.54–0.87 μm thick (Table 3). The perithallial SC was formed by one layer up to 2.94 μm in maximum thickness, composed of rod-shaped crystallites perpendicular to the cell membrane (Fig 1E; Table 3). The internal surface delimiting the cell lumen showed a granular calcification. In transverse section, it was observed an IC composed of irregular, more or less blocky granules (DB867; Fig 2C and 2D).

The primary hypothallus, i.e. the first algal cells growing directly over the substrate, was not detectable in our samples, but some prostrate cells growing over a conceptacle roof were visible, forming a secondary hypothallus. Here, the hypothallial primary crystallites appeared as packed regular and thin rhomboids, 0.4 x 0.4 ± 0.1 μm in size, which clearly progressed between two adjacent cell filaments (Figs 1H, 2E and 2F). Depending on the visual perspective, the same thin rhomboids showed only their thinnest dimension, thus appearing as rod-like structures parallel to the cell lumen, perfectly bordering the rounded shape of the outer cell wall (Fig 2E and 2F).

### Lithophyllum pseudoracemus

**Fig 3:**
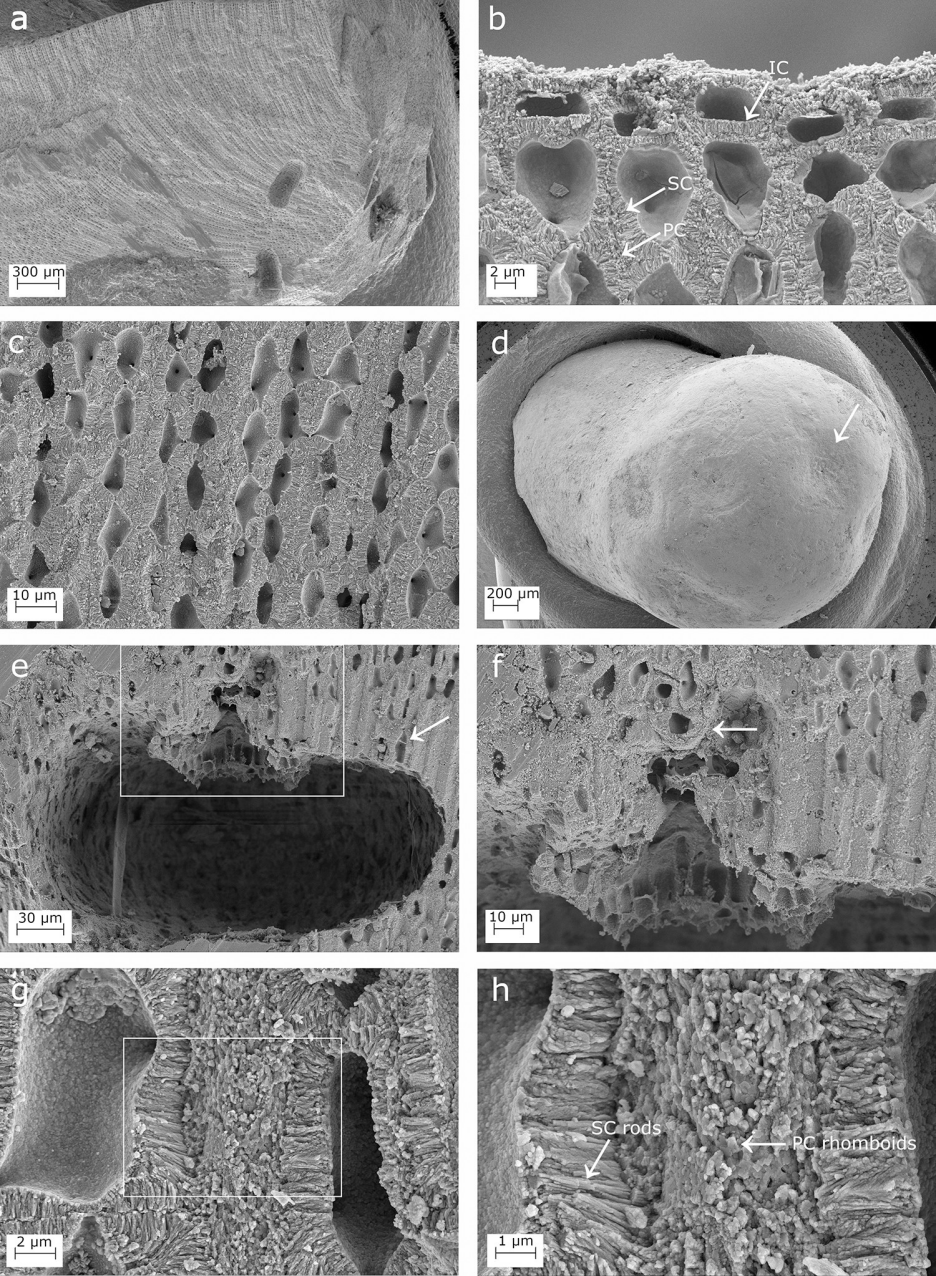
SEM images showing longitudinal sections of *Lithophyllum pseudoracemus*. (a) A protuberance longitudinally cut in half showing the fountain-like arrangement of perithallial cells, due to the diverging cell filaments toward the thallus surface; (b) epithallial cells showing the primary calcification (PC), the secondary calcification (SC), and the innermost calcified layer (IC); (c) the perithallus; (d) depressed conceptacle in surface view (arrow); (e) conceptacle chamber with no calcified columella and a trichocyte on the right (arrow); (f) magnification of the inset in (e) to show secondary hypothallial cells (arrow) over the pore canal; (g) the orientation of the PC crystallites is normal to those of the SC; (h) magnification of the inset in (g) to show the perithallial rhomboidal crystallites of the PC and the rod-shaped crystallites of the SC (arrows).

Microscopic anatomy: specimens were pink in colour, non-geniculate and fruticose with protuberances up to 5 mm long (up to 2 mm in DB768). In sample DB835, it was observed the same “fountain” pattern in the arrangement of perithallial cells, as already observed in *L*. *racemus* (Fig 3A). The perithallial banding was not detected. One or two epithallial cells were terminating each cell filament (Fig 3B), L 3.84–5.21 μm long and D 9.03–10.07 μm (Table 2). The perithallial cells were rectangular in longitudinal section, with numerous secondary pit-connections, L 15.40–16.28 μm and D 10.55–10.79 μm (Table 2; Fig 3C). The external surface of the asexual conceptacles appeared depressed, with a sunken pore aperture (Fig 3D). The asexual uniporate conceptacles in sample DB835 had a rounded chamber corresponding to a D/H ratio of 2.11 (Fig 3E; Table 2). The triangular pore canal was 42 μm long, counting 3 cells in the pore canal filament (Fig 3F; Table 2). No calcified columella was observed (Fig 3E). Trichocytes were observed exclusively at the periphery of a conceptacle chamber (Fig 3E). Sample DB768 was sterile.

Calcification: the epithallial cells were highly calcified with a cell wall area of 24.96–25.01 μm^2^ (Table 3). The PC was not detectable between the epithallial cell and the underlying meristem cell in the same filament, while the PC between two adjacent cell filaments was highly calcified and consisted of irregular granules (Fig 3B). The epithallial SC reached 0.98 μm in maximum thickness and was characterized by 1–2 layers of rods with granular texture laying perpendicular to the cell membrane (Fig 3B; Table 3). The internal surface delimiting the cell lumen showed a granular calcification, forming an IC between the plasma membrane and the perpendicular rods of the SC (Fig 3B).

**Fig 4:**
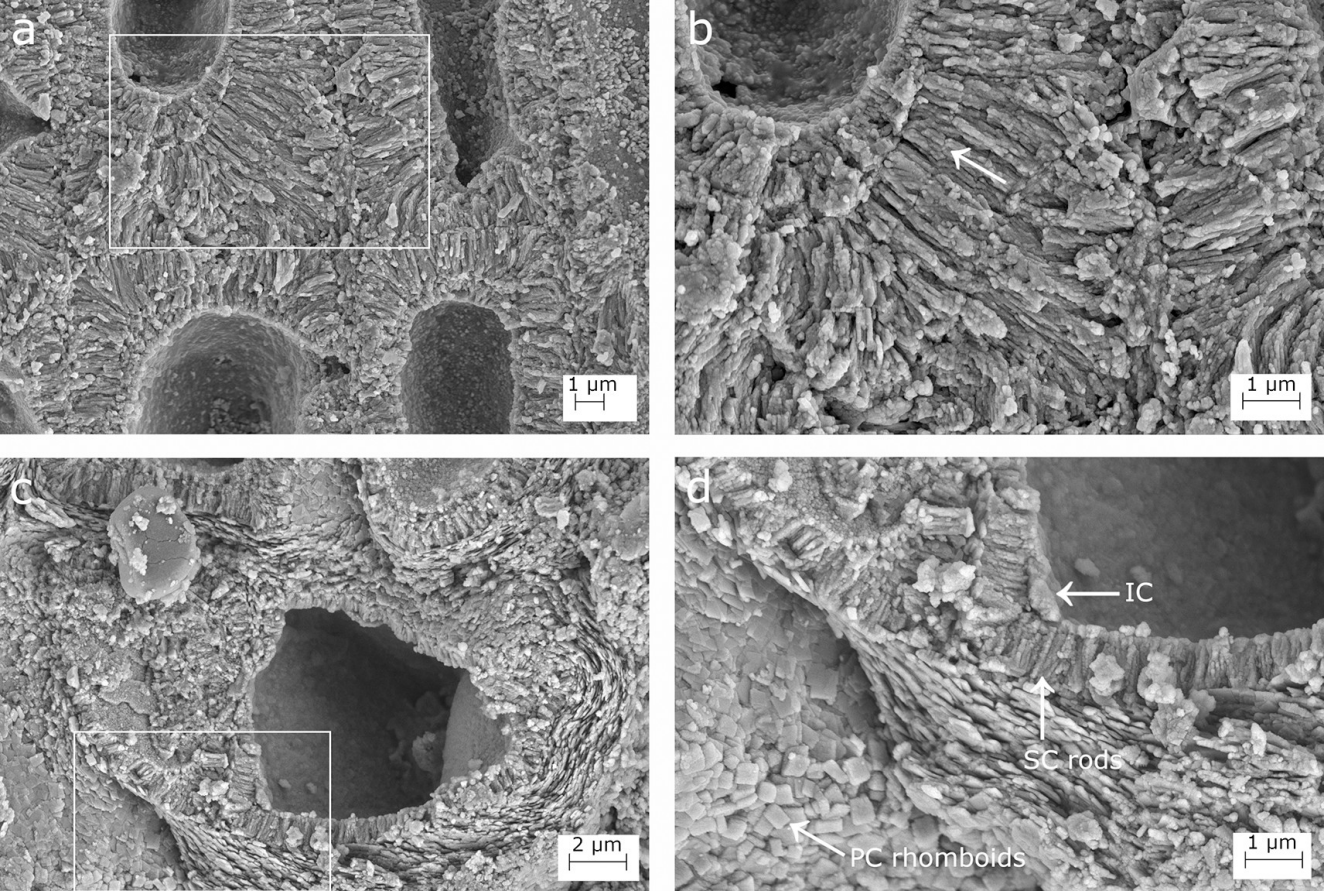
SEM images showing longitudinal sections of the calcified thallus in *Lithophyllum pseudoracemus*. (a) Multiple layers of secondary calcification forming the thick cell walls; (b) magnification of the inset in (a); the arrow points to the contact of two layers of SC; (c), secondary hypothallial cells in the proximity of the pore canal in Fig 3F; (d) magnification of the inset in (c) to show the rhomboidal crystallites in the hypothallial primary calcification (PC); the SC rods and the innermost calcified layer (IC).

The perithallus had oblong cells with strongly calcified cell walls (Fig 3C). The perithallial cell walls had an area of 99.71–100.48 μm^2^ (Table 3). The perithallial PC was 0.95–1.55 μm in thickness (Table 3), composed of packed irregular rhomboids with smooth indented margins (Fig 3G and 3H). The perithallial SC was 2.31–2.53 μm in maximum thickness, consisting of long perpendicular rods with a granular texture, arranged in 1 to 4 layers (Figs 3H and 4A, 4B; Table 3).

Over a conceptacle roof, in proximity to the pore canal, some prostrate cells of a secondary hypothallus clearly showed their PC (Fig 3F). This consisted of regular packed rhomboids, 0.4 x 0.4 ± 0.1 μm in size (Fig 4C and 4D). The rhomboids appeared as thin slips in some areas. The IC was detectable as a granular layer at the inner boundary of the SC (Fig 4D).

**Fig 5:**
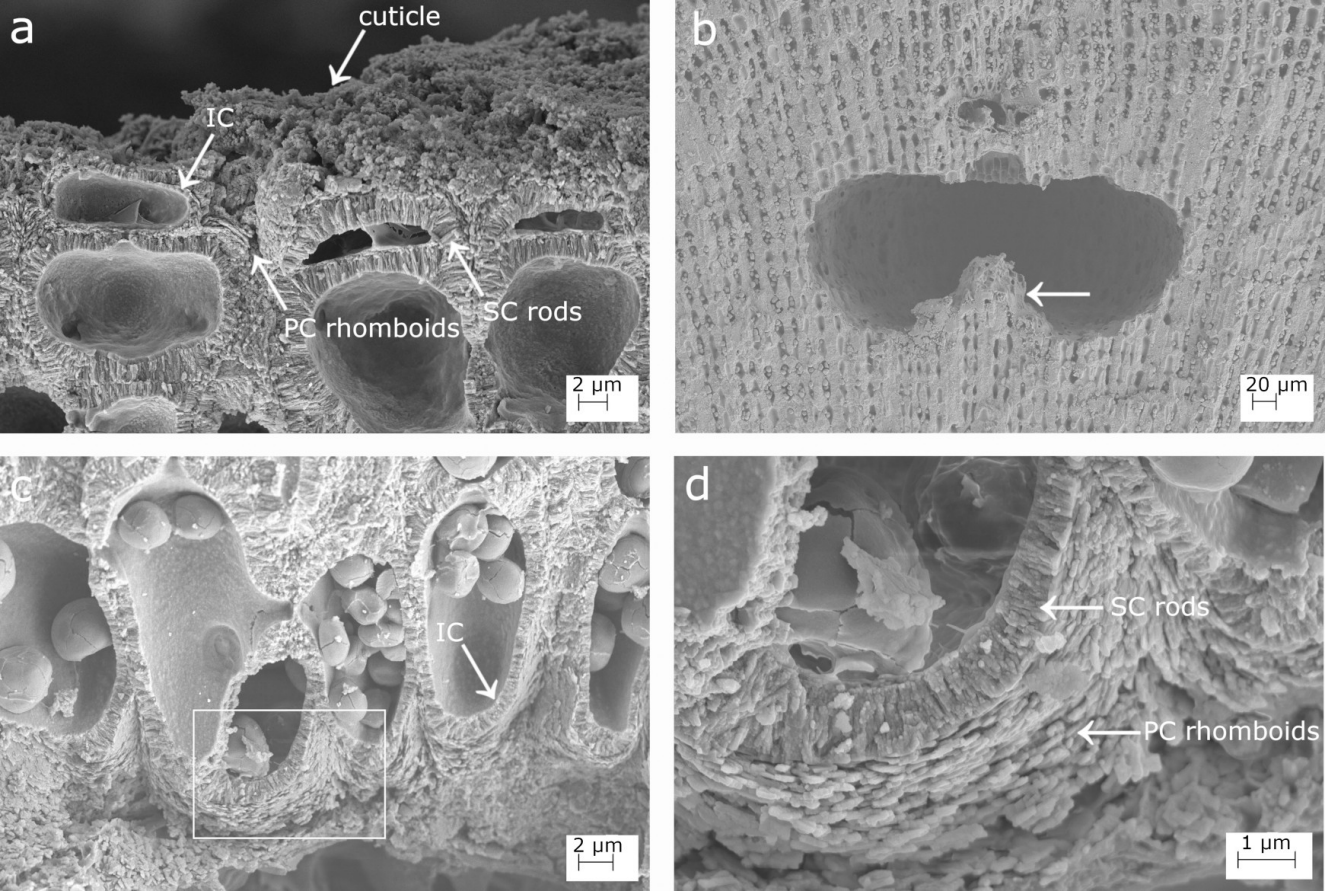
SEM images showing longitudinal sections in *Lithophyllum* cf. *racemus* DB865. (a) Epithallial calcified cells covered by a thick cuticle, and bordered by a primary calcification (PC), a secondary calcification (SC), and an innermost calcified layer (IC); (b) a conceptacle with a well-defined calcified columella (white arrow); (c) secondary hypothallial cells in the proximity of a pore canal and the innermost granular IC; (d) magnification of the inset in (c) showing the SC rhomboidal crystallites, and the PC rhomboids.

***Lithophyllum* cf. *racemus*.** Microscopic anatomy: specimen DB865 was pink in colour, non-geniculate and fruticose with protuberances up to 3 mm long. At small scale, it was visible the “fountain” pattern of the perithallial growth and in some areas also the banding pattern. One or 2 flattened epithallial cells were observed, L 4.78 ± 1.0 μm and D 13.12 ± 1.6 μm (Fig 5A; Table 2). The perithallial cells were rectangular in longitudinal section L 20.52 ± 2.5 μm and D 12.51 ± 2.5 μm (Table 2). Trichocytes were not observed. The asexual uniporate conceptacle had a kidney-shaped chamber D 325.13 μm and H 136.94 μm (Fig 5B; Table 2), equivalent to a D/H ratio of 2.37. The conceptacle chamber had an evident, calcified columella, reducing the conceptacle chamber height h1 to less than H/2 [59]. The pore canal was triangular in longitudinal section, 37.5 μm long, lined by 3 cells, with a cavity on top (Fig 5B; Table 2).

Calcification: the epithallial cells were strongly calcified and their surface appeared covered by a thick cuticle (Fig 5A). The epithallial cell wall area was 35.80 ± 12.0 μm^2^ (Table 3). The PC between epithallial cells of the same filament was undetectable or absent. The thick epithallial PC between adjacent cell filaments consisted of rhomboidal elements (Fig 5A). The epithallial SC reached a maximum thickness of 1.05 ± 0.2 μm and consisted of perpendicular rods (Fig 5A; Table 3), slightly more regular in shape than those in the perithallial SC. The internal surface delimiting the cell lumen was made of granules which constituted a very thin IC (Fig 5A).

The perithallus had oblong calcified cells with a cell wall area of 137.79 ± 29.2 μm^2^ (Table 3). The PC between two adjacent cell filaments, 1.28 ± 0.3 μm thick (Table 3), was composed of small irregular granules. The PC between cells of the same filament was inconspicuous. The SC had a maximum thickness of 2.87 ± 0.6 μm and consisted of a single layer of rods oriented perpendicular to the cell membrane (Table 3).

The secondary hypothallus was detected as prostrate cells growing over a conceptacle roof, close to the pore canal (Fig 5C and 5D). These hypothallial cells had a PC consisting of packed rhomboids, 0.3 x 0.3 ± 0.1 μm in size, running parallel to the cell membrane (Fig 5C and 5D). The hypothallial SC was composed of thin granular rods perpendicular to the cell membrane (Fig 5D). The IC in contact with the SC appeared granular from inside the cell lumen (Fig 5C).

#### *Lithophyllum* cf. *pseudoracemus*

**Fig 6:**
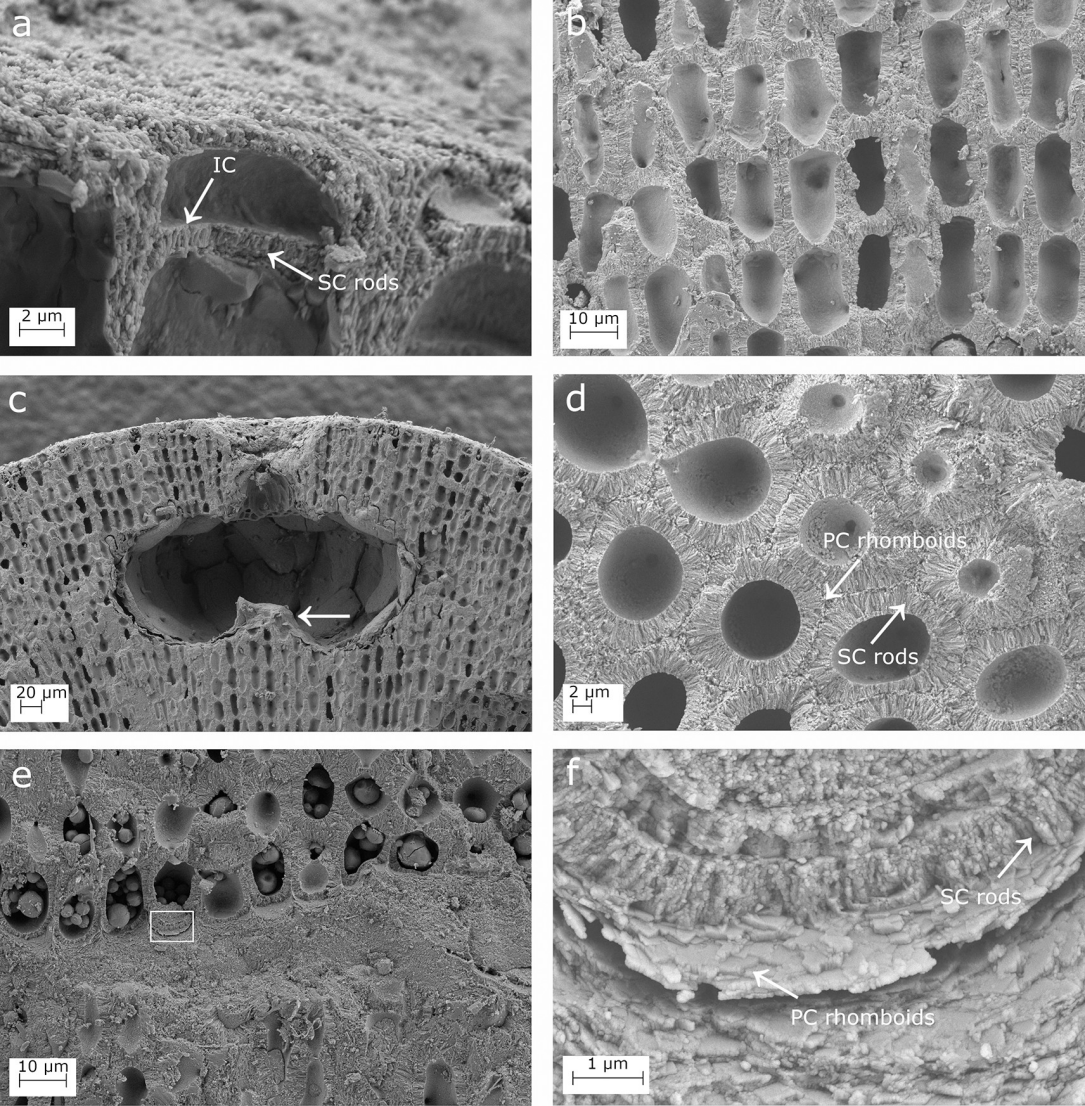
SEM images showing longitudinal sections of *Lithophyllum* cf. *pseudoracemus* DB866. (a) Epithallial cell bordered by the rods of the secondary calcification (SC), and the innermost calcified layer (IC); (b) the calcified perithallus; (c) a conceptacle with a short calcified columella (arrow); (d) transverse section showing the SC and the primary calcification (PC); (e) the primary hypothallus growing over the substrate; (f) magnification of the inset in (e) showing multiple layers of hypothallial PC rhomboids and SC rods.

Microscopic anatomy: specimen DB866 was dark pink in colour, non-geniculate and fruticose with protuberances up to 4 mm long. One or 2 epithallial cells were flattened, L 5.18 μm and D 11.31 μm (Fig 6A; Table 2). The perithallial cells were L 21.11 ± 2.5 μm long and D 11.82 ± 1.8 μm (Fig 6B; Table 2). The perithallial zonation with the banding pattern was not visible. The asexual uniporate conceptacles had a kidney-shaped chamber D 318.95 ± 20.3 μm, H 141.96 ± 11.4 μm (Fig 6C; Table 2) and 2.25 in D/H. In some conceptacles, a calcified columella was observed rising from the conceptacle floor (Fig 6C). The columella was variably developed, but its height never exceeded half of the height of the conceptacle chamber, resulting in h1>H/2. The pore canal was triangular, about 47 μm long, composed of 2–3 cells, with a rhomboidal cavity on top (Fig 6C; Table 2).

Calcification: the epithallus was strongly calcified (Fig 6A). The cell wall area was 27.18 ± 8.3 μm^2^ (Table 3). The PC between the epithallial and the subtending meristematic cells was undetectable, while the PC between adjacent cell filaments was thick and formed by irregular elements. The epithallial SC consisted of regular rods perpendicular to the cell membrane, reaching 0.99 ± 0.4 μm of maximum thickness (Fig 6A; Table 3). The cell lumina were delimited by the cell membrane which prevented the observation of the calcification (Fig 6A). Nevertheless, there was the evidence of an IC of granular elements in contact with the SC.

The perithallial cells were oblong and regularly arranged in rows. The cell walls had 117.68 ± 26.0 μm^2^ of area (Table 3). The PC between cells of the same filament was inconspicuous. The perithallial PC between cells of two adjacent filaments was 1.24 ± 0.3 μm thick and consisted of irregular elements which ran parallel to the cell lumen (Table 3). The perithallial SC consisted of a single layer of rods oriented perpendicular to the cell membrane, 2.43 ± 0.5 μm in maximum thickness (Table 3). The internal surface delimiting the cell lumen was granular, forming a clearly detectable IC layer in contact with the perpendicular rods of the SC. In transverse section, the perithallial cells had a clear hexagonal perimeter (Fig 6D).

The primary hypothallus had a calcification similar to the cells of the secondary hypothallus observed in the samples described above (Fig 6E and 6F). The PC was formed by packed rhomboidal slips bordering the cell (Fig 6F). The SC consisted of perpendicular rods arranged in 1 or 2 rows, very granular in texture.

### Statistics

**Fig 7:**
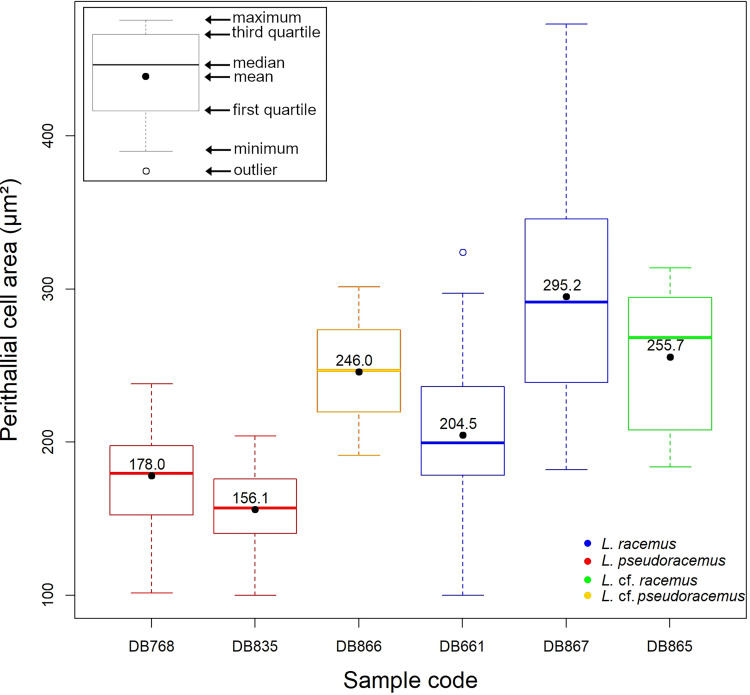
Box plot with the results of the perithallial cell area measurement. The numbers inside the plots indicate the mean values.

The statistical analyses ran on biometrical features highlighted significant differences among the perithallial cell sizes (p<<0.001; S1 Table, Fig 7). Particularly, the perithallial cells from both samples of *L*. *pseudoracemus* (DB768, DB835) were significantly smaller than those in *L*. *racemus* DB867 and the two non-molecularly classified DB865 and DB866 (p<<0.001; S1 Table; Fig 7). Instead, the *L*. *racemus* neotype (DB661) showed intermediate perithallial cell size (Fig 7), differing significantly from the other samples (p<0.01), with the exception of *L*. *pseudoracemus* DB768 (p = 0.23) (S1 Table; Fig 7). Other measured biometrical variables did not highlight further distinction between *L*. *pseudoracemus* and *L*. *racemus*.

**Fig 8:**
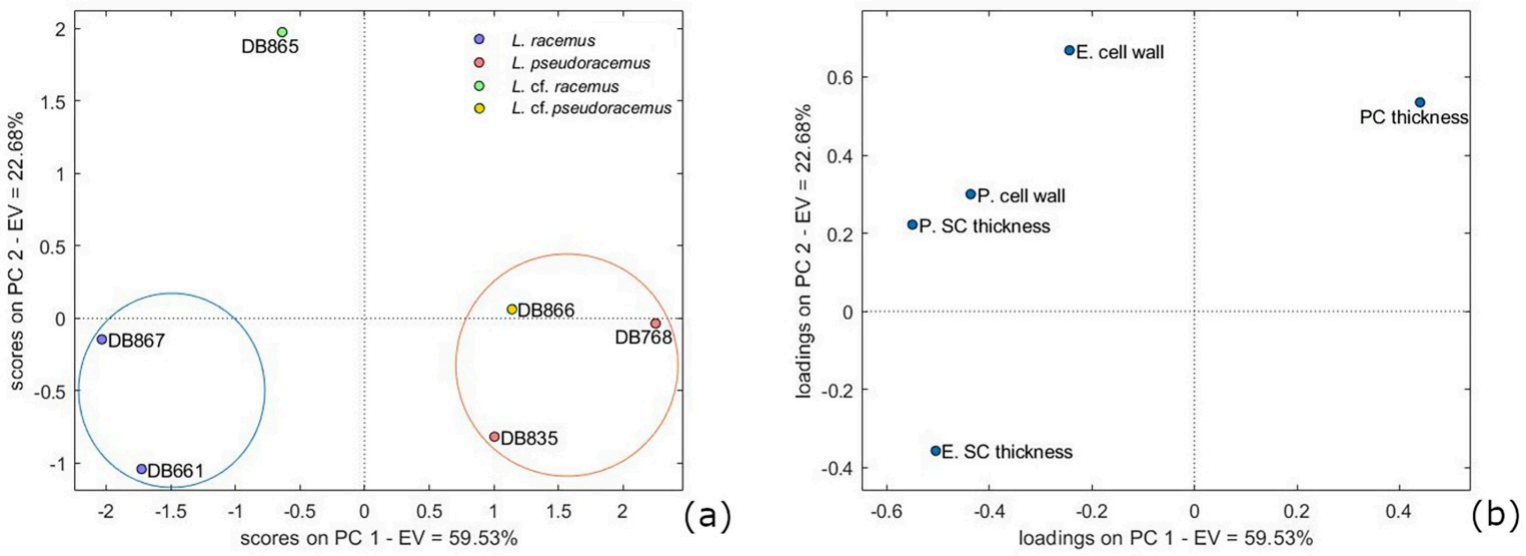
Principal component analysis (PCA). Score plot is shown in (a); loading plot of PCA on calcification data in both perithallial (P.) and epithallial (E.) cells (b). The plots model 83% of the total data variance. Variance proportions are shown along each component axis. Calcification types are evidenced with circles and classified in *L*. *racemus* type and *L*. *pseudoracemus* type.

The multivariate analysis on calcification traits was the only statistical test supporting a significant distinction between *L*. *racemus* and *L*. *pseudoracemus*. In the PCA model, the first two principal components (*PCs*) resolved the 83% of the total data variance (Fig 8). The main trend expressed on the *PC-1* axis was the increasing SC thickness with increasing cell wall area, expressed as high negative *PC-1* loadings for the perithallial cell wall area and the SC thickness (Fig 8). At the same time, the PC thickness tended to increase as the SC became thinner, as shown by the high positive *PC-1* loadings. The *PC-2* axis displayed positive loadings of the cell wall area, the perithallial PC thickness and SC thickness, as well as high negative loadings of the epithallial SC thickness (Fig 8). Overall, from the PCA, it was possible to distinguish two main calcification types based on natural groupings in the *PC-2* versus *PC-1* plot (Fig 8). The *L*. *racemus* type plotted with negative *PC-1* and negative *PC-2* scores (Fig 8) and was characterized by perithallial cells with high values for cell wall area and SC thickness (Fig 8; Table 3). The *L*. *pseudoracemus* type was characterized by positive *PC-1* and negative or neutral *PC-2* scores corresponding to perithallial cells with low values of cell wall area and thin SC. This category included also *L*. cf. *pseudoracemus* DB866. The sample *L*. cf. *racemus* DB865 differed from the other samples by having highly positive *PC-2* and negative *PC-1* scores (Fig 8). Since the *PC-1* scores explained 60% of the total data variance, they support the inclusion of *L*. cf. *racemus* DB865 in the *L*. *racemus* type. Most of the difference between sample DB865 and the other samples of the *L*. *racemus* type relied on the epithallial cell wall area, which was particularly high (Fig 8; Table 3).

**Fig 9:**
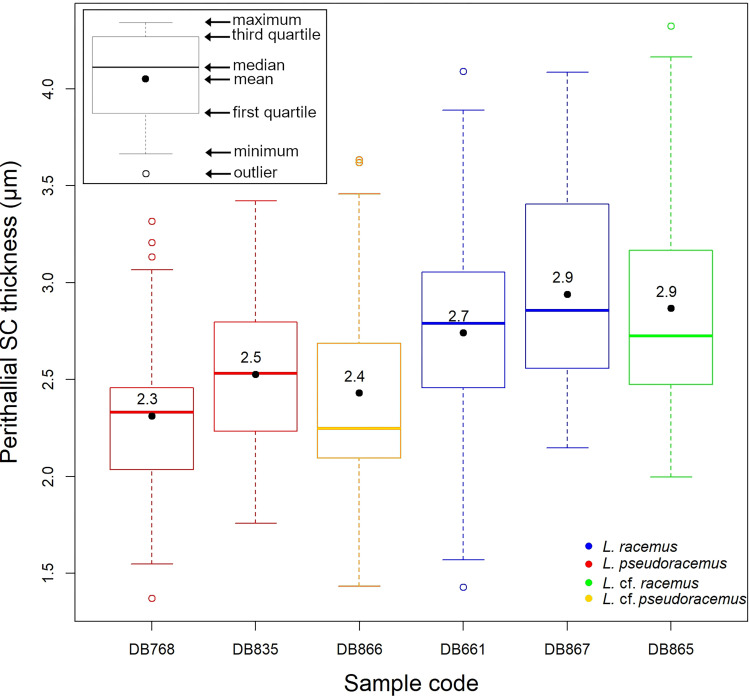
Box plot of the perithallial SC thickness. The numbers inside the plots indicate the mean values.

Given the results of the PCA, we explored the contribution of the considered variables in supporting the separation of *L*. *racemus* from *L*. *pseudoracemus*. The perithallial SC thickness was the only one to separate the two species with significant statistical support (Fig 9; S2 Table). Based on the PCA, *L*. cf. *pseudoracemus* DB866 was attributed to the *L*. *pseudoracemus* calcification type, while *L*. cf. *racemus* DB865 was attributed to the *L*. *racemus* calcification type (Figs 8 and 9; Table 2). The mean epithallial SC thickness was also different in *L*. *racemus* and *L*. *pseudoracemus* (Table 3), but the ANOVA results did not support a statistical significance of this feature.

**Fig 10:**
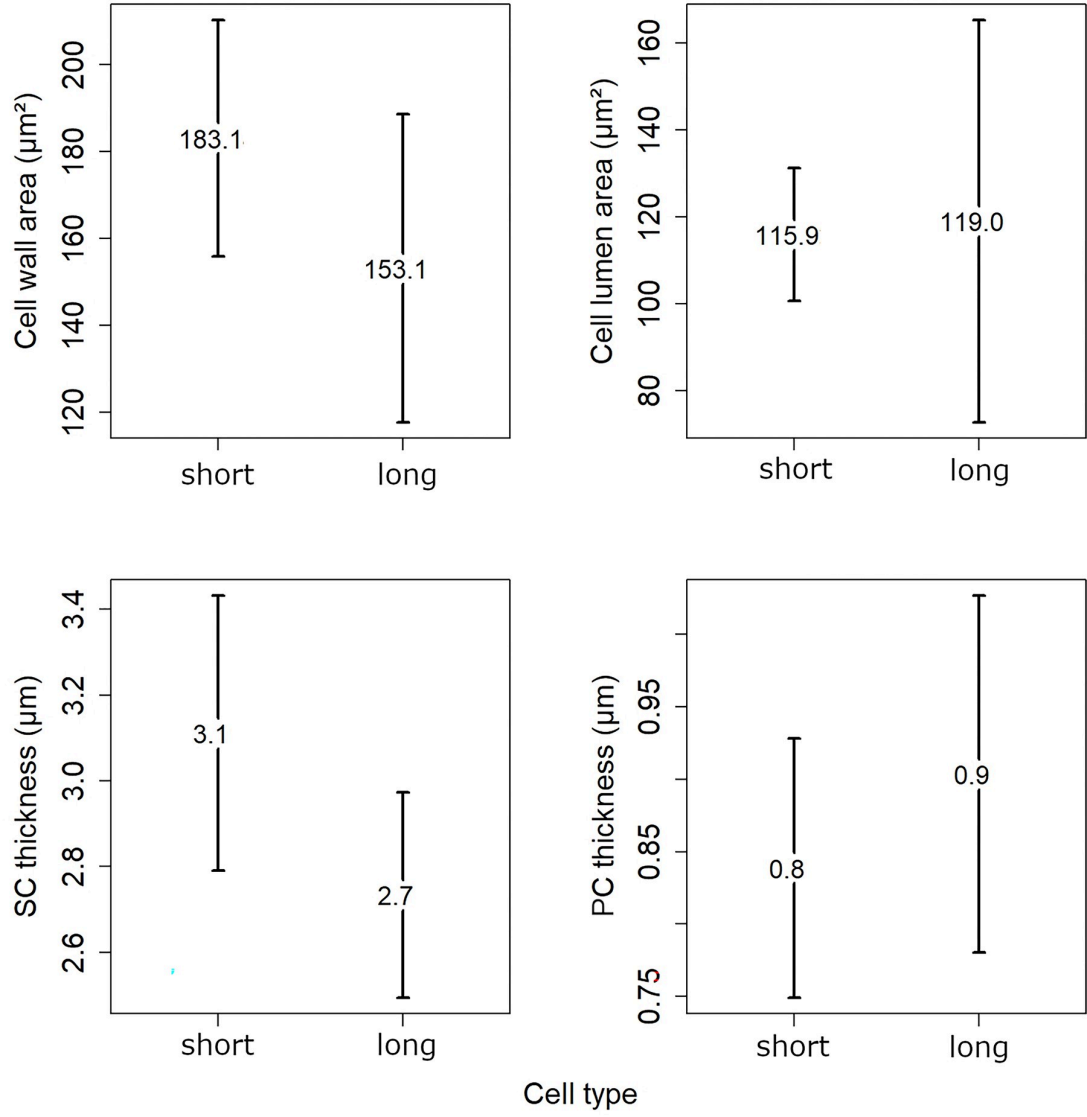
Comparison of mean values for short and long cells in *L*. *racemus* DB867. The numbers inside the plots indicate the mean values for the cell wall area, cell lumen area, SC thickness and PC thickness.

In *L*. *racemus* DB867, it was also possible to clearly distinguish between size of short and long cells within the perithallus (Fig 10). Long cells differed significantly from short cells by length (25.25 μm and 19.58 μm respectively, p<0.001). Short and long cells had very similar cell lumen area, but short cells had higher values of cell wall area and thicker SC, while long cells had thicker PC (Fig 10). Nevertheless, none of these differences were statistically significant (p > 0.05).

## Discussion

### Microscopic anatomy

At small scale, both *L*. *racemus* and *L*. *pseudoracemus* showed a “fountain” pattern in the arrangement of perithallial cells (Figs 1A and 3A), as already observed by [59] (pl. 63, Figs 5 and 6). In the samples of *L*. *racemus* DB661, DB867 and in *L*. cf. *pseudoracemus* DB865, the seasonal banding was also appreciable (Fig 1A).

The statistical analyses supported a significant difference between the size of perithallial cells of *L*. *racemus* and *L*. *pseudoracemus* (Fig 7; Tables 2, 4 and S1 Table; [65]). However, the molecularly identified samples of *L*. *racemus* show a very wide range of values of perithallial cell area (Fig 7; Table 2), thus jeopardizing the applicability of this single character for species separation.

The longitudinal medial section of the asexual conceptacle chambers of *L*. *pseudoracemus* was more rounded than that of *L*. *racemus*, having a lower D/H ratio (2.11 against 2.55). This pattern was confirmed also in the non-molecularly identified samples (2.25 in *L*. cf. *pseudoracemus* against 2.37 in *L*. cf. *racemus*). A calcified columella rising from the asexual conceptacle floor was observed in most samples but not in *L*. *pseudoracemus*, in agreement with [65]. The calcified columella rising from the conceptacle chamber floor of *L*. cf. *pseudoracemus* DB866 appeared variably developed, but its height was lower than half the height of the conceptacle chamber, consequently, h1 [59] was typically > H/2 (Fig 6C). The low number of observations, however, prevents any attempt of statistical analysis of this feature, that would deserve further investigation aimed at assessing its potential value.

Our biometrical analyses on molecularly characterized samples revealed some differences with the measures reported by [65]. In particular, we observed smaller conceptacles with a depressed surface in *L*. *pseudoracemus* (mean D 261.08 x H 123.75 μm *vs*. D 321.25 x H 175.00 μm) reported by [65] (Table 2). Moreover, we observed 3–5 layers of superposed epithallial cells in *L*. *racemus* instead of 1–2 as reported by [65]. These observations demonstrated high variability in microanatomy among samples and even within the same specimen, which would prevent a reliable distinction of the two species solely based on microanatomy and biometrical analysis, in agreement with [65]. Trichocytes have been demonstrated in the genus *Lithophyllum* only recently [71], but their mode of development hampers their identification under SEM and in petrographic thin sections. Nevertheless, no trichocyte was observed even in decalcified histological sections of *L*. *racemus*, while trichocytes were rarely detected in *L*. *pseudoracemus* by [65]. Despite the active search for trichocytes in our investigations, we were able to identify one single trichocyte exclusively in proximity of a conceptacle chamber in *L*. *pseudoracemus* (Fig 3E), thus confirming the published report [65].

### Calcification traits

The suitability of skeletal ultrastructure for the taxonomy of calcareous red algae has been proved only recently [38, 39, 70]. Auer and Piller [38] introduced the morphology of crystallites in the PC of epithallial and meristem cells as a new morphological criterion to discriminate among different clades. Among the subfamily Lithophylloideae, *Lithophyllum incrustans*, *L*. *kotschyanum*, *L*. *lichenoides*, *L*. *pygmaeum* and *L*. *byssoides* (as *Titanoderma byssoides*) were analysed by [38]. According to these authors, the defining traits of Lithophylloideae are rhombic plates as dominant morphotype of epithallial cell wall crystallites and perpendicular rod-shaped crystallites in the SC [38]. Our observations are in substantial agreement, reporting rhomboid-shaped crystallites in the PC and perpendicular rod-shaped crystallites in the SC in both *L*. *racemus* and *L*. *pseudoracemus*, as already concluded by a deep learning approach on SEM images of the two species [70]. However, we found evidence of three different calcified layers in *L*. *racemus* and *L*. *pseudoracemus*: the primary outer calcification formed by parallel rhomboids (PC); the median one with perpendicular rods of the secondary calcification (SC) and the innermost granular layer (IC) (Figs 1C and 4D). The IC appeared composed of granular elements (Fig 1C), was located between the plasma membrane and the SC, and was often covered by organic residue of the plasma membrane. Moreover, we described regular and obvious rhomboids in the hypothallial cells of both primary and secondary hypothallus (Figs 2E, 2F, 4C, 4D, 5C, 5D, 6E and 6F), while they were much less regular in the other cells, regardless of the sample. In fact, within the perithallus, the PC crystallites had irregular shapes and rhomboids became hardly distinguishable, while the crystallites of the SC remained visibly rod-shaped. The particularly regular PC rhomboids observed in the hypothallial cells were apparently absent between epithallial cells of the same filament, and not obvious elsewhere, except in the lateral (= between adjacent filaments) epithallial cell walls of *L*. cf. *racemus* DB865 (Fig 5A). In the meristem, both *L*. *racemus* and *L*. *pseudoracemus* shared a similar pattern of absence of PC and increasing SC thickness, in the newly formed horizontal cell wall that cuts off a new epithallial cell (Fig 1B). At the same time of the new epithallial cut-off, the lateral cell walls (between adjacent filaments) that contain the meristem cell and the epithallial cells issued from it, appeared as a pre-existing calcite “envelope” with well-developed and relatively homogeneous thickness (Fig 1B). In the congeneric species *Lithophyllum kotschyanum* the pattern of calcification of the new epithallial cells differs, since an increasing thickness of the SC toward the lower base of the meristem cell was observed [38]. This observation also supports a species-specific control of calcification traits in CCA. DNA sequencing coupled with the analysis of calcification traits on a broader range of species could elucidate the diagnostic potential of the pattern of calcification of the meristem cutting off the new epithallial cells.

The loss of definition in the primary crystallite shape observed in perithallial cells of *L*. *racemus* and *L*. *pseudoracemus* could be related to solution/precipitation or recrystallization processes and has been already reported in *L*. *kotschyanum* along with progressively increasing calcification [38].

Considering the measured calcification traits, the PCA results (Fig 8) succeeded in allowing the circumscription and separation of two groups of samples which included distinctively the genetically classified *L*. *racemus* (DB661, DB867) and *L*. *pseudoracemus* (DB768, DB835). Overall, *L*. *racemus* was characterized by perithallial cells with large cell wall area (145.48 μm^2^ by mean) and thicker SC (2.84 μm); *L*. *pseudoracemus* had instead small perithallial cell wall area (100.09 μm^2^ by mean) with thinner SC (2.42 μm) (Fig 8; Table 3). To note is also the direct proportion between the SC thickness and the cell wall area in the perithallus of our corallines (Fig 8). The *L*. *racemus* and *L*. *pseudoracemus* specimens analysed in this work were collected in the Western Mediterranean Basin. The specimens *L*. *racemus* DB867 and *L*. *pseudoracemus* DB768 occurred sympatrically and at a similar depth (Table 1) and nevertheless showed a highly significant distinction in terms of calcification traits (Fig 8). In particular, DB867 had thin PC and high values of SC thickness and cell wall area, while DB768 had thick PC, thin SC and small cell wall area (Fig 8; Table 3).

Samples *L*. cf. *racemus* DB865 and *L*. cf. *pseudoracemus* DB866, non-molecularly identified, were collected in the Eastern Mediterranean, where the presence of both *L*. *racemus* and *L*. *pseudoracemus* was also confirmed by molecular techniques [65]. According to the PCA, sample DB866 fell in the *L*. *pseudoracemus* calcification type (Fig 8). Interestingly, samples with thick perithallial SC had also thick SC in the epithallial cells (Table 3). The epithallial cells in specimen DB865 had the largest diameter (Fig 5A; Table 2), explaining the high cell wall area despite the relatively thin SC. In the perithallus, its cell wall area was large and the SC was thick, in agreement with the major defining traits of *L*. *racemus* (Fig 8; Table 3) and actually the *PC-1* axis of the PCA, which explained most of the data variance (60%), supports the inclusion of *L*. cf. *racemus* DB865 in the *L*. *racemus* calcification type (Fig 8). This specimen was collected at the shallowest depth (10 m), while all the other specimens were collected deeper than 40 m, rising a question about the possible response of the epithallial cell size to light availability or other still undefined oceanographic controls.

The distinct zonation (= banding) of *L*. *racemus* DB867 provided an opportunity to focus on calcification traits in short and long cells separately (Fig 10). In literature, short cells are reported to show thicker cell walls and lower Mg/Ca ratio compared to long cells [33, 37]. Our results showed that *L*. *racemus* short perithallial cells possess higher SC thickness, and therefore a larger cell wall area, in comparison with long perithallial cells, although keeping an almost stable cell lumen area (Fig 10). The PC observed between two cells of adjacent filaments was thicker in long cells produced in the warm months, while short cells had larger cell wall area due to the thicker SC (Fig 10) [39]. Therefore, we could speculate that warmer temperatures and/or longer light periods would have triggered the production of primary crystallites. The low values of PC thickness in short cells might be explained by a SC that could grow at the expense of the PC during the cold months. This hypothesis is supported by the observation of an inverse correlation between SC and PC thickness, observed in the perithallial cells of most samples (Fig 8; Table 3).

### Biomineralization

The calcification in coralline red algae had been traditionally considered a controlled process, by means of cell wall mineralization [72–74]. This view was questioned by the report of different Mg/Ca ratios associated to distinct anatomical structures in CCA [75] and in particular, of higher-Mg calcite detected in wound repairs, in the primary calcification and in hypothallial cells of *Phymatolithon* and *Clathromorphum* [18, 76]. Based on this considerable Mg/Ca ratio variation observed in the Mg-calcite within single specimens, and on the observed relationship of Mg/Ca with ambient temperature, a biologically induced origin of CCA calcification was suggested [76]. The biologically induced mineralization is defined as the secondary precipitation of minerals resulting from interactions between the biological activity and the environment [77].

In contrast with the idea of a biologically-induced calcification in CCA [76] it was recently demonstrated that the ultrastructural shape and arrangement of crystallites in the cell walls is shared by species belonging to the same phylogenetic clade [38, 39]. Moreover, [39] showed the occurrence of species-specific distinct styles of PW calcification within the genus *Lithothamnion*. Despite the variable and quite distinct cell size in *L*. *racemus* and *L*. *pseudoracemus*, the ultrastructures observed and analysed in this paper confirm the recently described lithophylloid pattern [38], and the phylogenetic control on the calcification process and on the morphology and orientation of the produced crystallites (Fig 8).

Raz et al. [78] experimentally demonstrated that high-Mg calcite forms via an amorphous precursor phase, composed of spherical particles. Similar findings have been recorded later in corals [79], where amorphous calcium carbonate (ACC) precursors precipitate before being transformed into crystalline calcium carbonate (CCC) after about a day. Similar amorphous-to crystalline pathways were reported in crustaceans, annelids, and mollusk larval shells [80]. From this perspective, the inner layer of calcified grains observed in our corallines (Fig 1C) could represent an intermediate precursor phase preceding the formation of the rod-shaped crystallites. The IC is the only calcification occurring just after the cut off of a new epithallial cell from the meristem, and we frequently observed multiple layers of perpendicular rods within the SC (Fig 4A). Therefore, we suggest that IC would likely be the first step in the formation of SC, with SC multiple-layer thickening deriving from a deposition of a new layer after reactivation of IC. From this point of view, a CCA calcification proceeding from the inner part of the cell wall towards its periphery appears the only plausible explanation [76].

The Mg/Ca ratio in CCA is variable not only among specimens or within a single specimen. At a higher detail, NanoSIMS analysis of the Mg/Ca ratios in *L*. *glaciale* showed higher values in summer cells, with a pronounced variation within a single cell wall, both in the natural environment and in controlled conditions with constant temperature, although at a lesser extent [37]. This observation would suggest that the Mg/Ca ratio is controlled primarily by the temperature of the environment, as assessed across phyla at a global scale [81], but also that the living CCA cells mediate the calcite Mg/Ca ratio at the ultrastructural scale. In the perithallial cells of *L*. *glaciale* the highest ratios were recorded in the PC, and at the boundary of the cell lumen, with NanoSIMS evidence of further high Mg/Ca values, localized concentrically in the SC [37] (Fig 2A). This pattern of concentric higher Mg/Ca values supports the hypothesis of successive activations of the IC, possibly driving the formation of additional SC crystallite layers.

Interestingly, we observed the best developed calcite rhomboids in the PC among adjacent cell filaments and in the hypothallial cell walls, both in the primary and secondary hypothallus. We observed also thicker PC in longer cells, which explains the occurrence of higher Mg/Ca ratio in the cell wall of longer perithallial cells produced during the warm season [25, 34, 37, 76]. These observations support the hypothesis that the primary and secondary calcification may be controlled by distinct processes: the calcification of the actively (fast?) developing anatomical parts (hypothallial cells, wound repairs), and in particular the PC and the IC, could be mediated by a higher Mg/Ca phase controlled by distinct physiological activity of CCA cells and their organic templates (e.g.: [15, 18]; on the contrary, the calcification of the SC would develop from the IC and partially at the expenses of the PC, by incremental growth of one or multiple generation of crystallites with relatively lower Mg/Ca ratio.

## Conclusions

We used SEM image analyses to compare the classical biometrical descriptors and the calcification traits of two morphologically similar, but genetically distinct sympatric species, *L*. *racemus* and *L*. *pseudoracemus*. The perithallial cell area differed significantly between *L*. *racemus* and *L*. *pseudoracemus* and the shape of the asexual conceptacles together with the occurrence and shape of the calcified columella could suggest a morphoanatomical criterion for species identification, however, their use would be hampered by a non-negligible degree of variability and values superposition. Therefore, a morphological identification solely based on biometrical measurements of cells and reproductive structures confirmed to be ineffective for species discrimination of *L*. *racemus* from *L*. *pseudoracemus*.

The morphology of crystallites in the high-Mg calcite cell walls was coherent with previous studies on other species belonging to the same clade. We observed no distinction of crystallite shape and organization at species level. We rather found differences in the degree of cell calcification, and we could distinguish two *L*. *racemus* and *L*. *pseudoracemus* calcification types from a multivariate analysis accounting for the SC thickness, the PC thickness and the cell wall area. Most of the differences were explained by the perithallial SC thickness, higher in *L*. *racemus* and lower in *L*. *pseudoracemus*. One corollary is that the exclusive analysis of the cell-wall ultrastructures in epithallial and meristematic cells is a valid diagnostic criterion for higher taxa, but other elements must be taken into account for the identification of congeneric and very similar specimens.

Evidence from SEM images showed the presence of a granular calcified layer in the innermost part of the cell wall (IC), in addition to the SC and PC known in the literature. We suggest that this could represent the precursor phase in the formation of secondary crystallites, becoming an important clue of the biomineralization mechanism in CCA.

A comparative analysis of the calcification of short and long cells, in the framework of published reports about the pattern of variation in the Mg/Ca ratio, suggested that cell elongation both in the perithallus and in the hypothallus is associated with the enhanced production of PC with higher Mg/Ca ratio, possibly triggered by warmer temperatures and/or longer light periods. Moreover, the inverse proportion between perithallial SC and PC thickness, together with the absence of PC in the newly formed wall cutting off an epithallial cell from the meristem, support the existence of two different pathways for the formation of PC and SC.

Our findings suggest the existence of a taxon-dependent hierarchy of ultrastructural patterns and calcification traits, still largely unexplored, consistent with the concept of biologically-controlled calcification in coralline red algae. Classical morphoanatomy should be integrated with new characters such as skeletal ultrastructures, to be assessed in genetically identified specimens, with the aim to improve rather than neglect the information about phylogeny, functions, adaptations and ecology, conveyed by anatomical structures and organization patterns.

## Supporting information

S1 Table Results of statistical tests. Testing the difference of the perithallial cell area of *L*. *racemus* (DB661, DB867), *L*. *pseudoracemus* (DB768, DB835), *L*. cf. *racemus* DB865, and *L*. cf. *pseudoracemus* DB866. Statistically significant p-values are given in bold. Kruskal-Wallis test significance at α = 0.05; Dunn’s test significant at p ≤ α/2.
(DOCX)

S2 Table Results of statistical tests. Testing the differences of the perithallial SC thickness in *L*. *racemus* (DB661, DB867), *L*. *pseudoracemus* (DB768, DB835), *L*. cf. *racemus* DB865, and *L*. cf. *pseudoracemus* DB866. Statistically significant p-values are given in bold. ANOVA test significance at α = 0.05; Tukey’s test significant at p ≤ α.
(DOCX)

## References

[1] Freiwald A, Henrich R. Reefal coralline algal build-ups within the Arctic Circle: morphology and sedimentary dynamics under extreme environmental seasonality. Sedimentology. 1994;41:963–984.

[2] Ballesteros E. Mediterranean coralligenous assemblages: a synthesis of present knowledge. Oceanogr Mar Biol. 2006;44:123–195.

[3] Bressan G, Babbini L, Ghirardelli L, Basso D. Bio-costruzione e bio-distruzione di Corallinales nel mar Mediterraneo. Biol Mar Medit. 2001;8:131–174.

[4] Caragnano A, Colombo F, Rodondi G, Basso D. 3-D distribution of nongeniculate Corallinales: a case study from a reef crest of South Sinai (Red Sea, Egypt). Coral Reefs. 2009;28:881–891.

[5] Amado-Filho GM, Moura RL, Bastos AC, Salgado LT, Sumida PY, Guth AZ, et al. Rhodolith beds are major CaCO_3_ bio-factories in the tropical south west Atlantic. PLoS One. 2012;7:e35171. 22536356 10.1371/journal.pone.0035171 PMC3335062

[6] Bracchi VA, Basso D, Marchese F, Corselli C, Savini A. Coralligenous morphotypes on subhorizontal substrate: a new categorization. Cont Shelf Res. 2017;144:10–20.

[7] Ingrosso G, Abbiati M, Badalamenti F, Bavestrello G, Belmonte G, Cannas R, et al. Mediterranean bioconstructions along the Italian coast. Adv Mar Biol. 2018;79:61–136. doi: 10.1016/bs.amb.2018.05.001 30012277

[8] Marchese F, Bracchi V, Lisi G, Basso D, Corselli C, Savini A. Assessing fine-scale distribution and volume of Mediterranean algal reefs through terrain analysis of multibeam bathymetric data. A case study in the southern Adriatic continental shelf. Water. 2020;12: doi: 10.3390/w12010157

[9] Cebrián E, Ballesteros E, Canals M. Shallow rocky bottom benthic assemblages as calcium carbonate producers in the Alboran Sea (southwestern Mediterranean). Oceanol Acta. 2020;23:311–322.

[10] Canals M, Ballesteros E. Production of carbonate particles by phytobenthic communities on the Mallorca-Menorca shelf, northwestern Mediterranean Sea. Deep Sea Res. 2007;44:611–629.

[11] Fortunato H, Schäfer P. Coralline algae as carbonate producers and habitat providers on the Eastern Pacific coast of Panama: preliminary assessment. Neues Jahrb Geol P-A. 2009;253:145–161.

[12] Basso D. Carbonate production by calcareous red algae and global change. In: Basso D, Granier B, editors. Calcareous algae and global change: from identification to quantification. Geodiversitas. 2012;34:13–33.

[13] Bracchi VA, Basso D. The contribution of calcareous algae to the biogenic carbonates of the continental shelf: Pontian Islands, Tyrrhenian Sea, Italy. Geodiversitas. 2012;34:61–76.

[14] Pomar L, Baceta JI, Hallock P, Mateu-Vicens G, Basso D. Reef building and carbonate production modes in the west-central Tethys during the Cenozoic. Mar Petrol Geol. 2017;83:261–304.

[15] Bilan MI, Usov AI. Polysaccharides of calcareous algae and their effect on the calcification process. Russ J Bioorganic Chem. 2001;27:2–16. 10.1023/a:1009584516443 11255640

[16] Morse JW, Andersson AJ, Mackenzie FT. Initial responses of carbonate-rich shelf sediments to rising atmospheric pCO_2_ and ‘‘ocean acidification”: role of high Mg-calcites, Geochim Cosmochim Ac. 2006;70:5814–5830.

[17] Rahman MA, Halfar J. First evidence of chitin in calcified coralline algae: New insights into the calcification process of *Clathromorphum compactum*. Sci Rep. 2014;4:6162. 25145331 10.1038/srep06162 PMC4141250

[18] Nash MC, Adey W. Multiple phases of Mg-calcite in crustose coralline algae suggest caution for temperature proxy and ocean acidification assessment: lessons from the ultrastructure and biomineralization in *Phymatolithon* (Rhodophyta, Corallinales). J Phycol. 2017;53:970–984. 28671731 10.1111/jpy.12559

[19] Smith AM, Sutherland JE, Kregting L, Farr TJ, Winter DJ. Phylomineralogy of the Coralline red algae: Correlation of skeletal mineralogy with molecular phylogeny. Phytochemistry. 2012;81:97–108. doi: 10.1016/j.phytochem.2012.06.003 22795764

[20] Aguirre J, Riding R, Braga JC. Diversity of coralline red algae: origination and extinction patterns from the Early Cretaceous to the Pleistocene. Paleobiology. 2000;26:651–667.

[21] Bracchi VA, Nalin R, Basso D. Paleoecology and dynamics of coralline dominated facies during a Pleistocene transgressive–regressive cycle (Capo Colonna marine terrace, Southern Italy). Palaeogeogr Paleoclimatol Palaeoecol. 2014;414:296–309.

[22] Bracchi VA, Nalin R, Basso D. Morpho-structural heterogeneity of shallow-water coralligenous in a Pleistocene marine terrace (Le Castella, Italy). Palaeogeogr Paleoclimatol Palaeoecol. 2016;454:101–112.

[23] Bracchi VA, Basso D, Savini A, Corselli C. Algal reefs (Coralligenous) from glacial stages: origin and nature of a submerged tabular relief (Hyblean Plateau, Italy). Mar Geol. 2019;411:119–132.

[24] Teichert S, Woelkerling WJ, Munnecke A. Coralline red algae from the Silurian of Gotland indicate that the order Corallinales (Corallinophycidae, Rhodophyta) is much older than previously thought. Palaeontology. 2019;62:599–613.

[25] Halfar J, Zack T, Kronz A, Zachos JC. Growth and high‐resolution paleoenvironmental signals of rhodoliths (coralline red algae): a new biogenic archive. J Geophys Res-Oceans. 2000;105:22107–22116.

[26] Hetzinger S, Halfar J, Zack T, Gamboa G, Jacob DE, Kunz BE, et al. High-resolution analysis of trace elements in crustose coralline algae from the North Atlantic and North Pacific by laser ablation ICP-MS. Palaeogeogr Palaeoclimatol Palaeoecol. 2011;302:81–94.

[27] Fietzke J, Ragazzola F, Halfar J, Dietze H, Foster LC, Hansteen TH, et al. Century-scale trends and seasonality in pH and temperature for shallow zones of the Bering Sea. P Natl Acad Sci. 2015;112:2960–2965. doi: 10.1073/pnas.1419216112 25713385 PMC4364235

[28] Ragazzola F, Caragnano A, Basso D, Schmidt DN, Fietzke J. Establishing temperate crustose Early Holocene coralline algae as archive for paleoenvironmental reconstructions of the shallow water habitats of the Mediterranean Sea. Paleontology. 2019;63:155–170.

[29] Cabioch J. Contribution à l’étude morphologique, anatomique et systématique de deux Mélobésiées: *Lithothamnium calcareum* (Pallas) Areschoug et *Lithothamnium corallioides* Crouan. Bot Mar. 1966;9:33–53.

[30] Basso D. Study of living calcareous algae by a paleontological approach: the non-geniculate Corallinaceae (Rhodophyta) of the soft bottoms of the Tyrrhenian Sea (western Mediterranean). The genera *Phymatolithon* Foslie and *Mesophyllum* Lemoine. Riv Ital Paleont Strat. 1995;100:575–596.

[31] Basso D. Living calcareous algae by a paleontological approach: the genus *Lithothamnion* Heydrich nom. cons. from the soft bottoms of the Tyrrhenian Sea (Mediterranean). Riv Ital Paleont Strat. 1995;101:349–366.

[32] Foster MS. Rhodoliths: between rocks and soft places. J Phycol. 2001;37:659–667.

[33] Kamenos NA, Cusack M, Huthwelker T, Lagarde P, Scheibling RE. Mg-lattice associations in red coralline algae. Geochim Cosmochim Ac. 2009;73:1901–1907.

[34] Caragnano A, Basso D, Jacob DE, Storz D, Rodondi G, Benzoni F, et al. The coralline red alga *Lithophyllum kotschyanum* f. *affine* as proxy of climate variability in the Yemen coast, Gulf of Aden (NW Indian Ocean). Geochim Cosmochim Ac. 2014;124:1–17.

[35] Flajs G. Skeletal ultrastructures of calcareous algae. Palaeontogr Abt B. 1977;160:69–128.

[36] Adey WH, Chamberlain YM, Irvine LM. An SEM-based analysis of the morphology, anatomy, and reproduction of *Lithothamnion tophiforme* (Esper) Unger (Corallinales, Rhodophyta), with a comparative study of associated North Atlantic arctic/subarctic Melobesioideae. J Phycol. 2005;41:1010–1024.

[37] Ragazzola F, Foster LC, Jones CJ, Scott TB, Fietzke J, Kilburn MR, et al. Impact of high CO_2_ on the geochemistry of the coralline algae *Lithothamnion glaciale*. Sci Rep. 2016;6:20572. 26853562 10.1038/srep20572 PMC4744931

[38] Auer G, Piller WE. Nanocrystals as phenotypic expression of genotypes—An example in coralline red algae. Science Advances. 2020;6 doi: 10.1126/sciadv.aay2126 32095524 PMC7015681

[39] Bracchi VA, Piazza G, Basso D. A stable ultrastructural pattern despite variable cell size in *Lithothamnion corallioides*. Biogeosciences. 2021;18:6061–6076.

[40] Bailey JC, Gabel JE, Freshwater DW. Nuclear 18S rRNA gene sequence analyses indicate that the Mastophoroideae (Corallinaceae, Rhodophyta) is a polyphyletic taxon. Phycologia. 2004;43:3–12.

[41] Kato A, Baba M, Suda S. Revision of the Mastophoroideae (Corallinales, Rhodophyta) and polyphyly in nongeniculate species widely distributed on Pacific coral reefs. J Phycol. 2011;47:662–72. 27021995 10.1111/j.1529-8817.2011.00996.x

[42] Pardo C, Lopez L, Peña V, Hernández-Kantún J, Le Gall L, Bárbara I, et al. A multilocus species delimitation reveals a striking number of species of coralline algae forming maerl in the OSPAR maritime area. PLoS One. 2014;9:e104073. doi: 10.1371/journal.pone.0104073 25111057 PMC4128821

[43] Hernández-Kantún JJ, Riosmena-Rodriguez R, Hall-Spencer JM, Peña V, Maggs CA, et al. Phylogenetic analysis of rhodolith formation in the Corallinales (Rhodophyta). Eur J Phycol. 2015;50:46–61.

[44] Caragnano A, Foetisch A, Maneveldt GW, Millet L, Liu LC, Lin SM, et al. Revision of Corallinaceae (Corallinales, Rhodophyta): recognizing *Dawsoniolithon* gen. nov., *Parvicellularium* gen. nov. and Chamberlainoideae subfam. nov. containing *Chamberlainium* gen. nov. and *Pneophyllum*. J Phycol. 2018;54:391–409. 29574890 10.1111/jpy.12644

[45] De Jode A, David R, Haguenauer A, Cahill A, Erga Z, Guillemain D, et al. From seascape ecology to population genomics and back. Spatial and ecological differentiation among cryptic species of the red algae *Lithophyllum stictiforme*/*L*. *cabiochiae*, main bioconstructors of coralligenous habitats. Mol Phylogenet Evol. 2019;137:104–113. 30951921 10.1016/j.ympev.2019.04.005

[46] Pezzolesi L, Peña V, Le Gall L, Gabrielson PW, Kaleb S, Hughey JR, et al. Mediterranean *Lithophyllum stictiforme* is a genetically diverse species complex: implications for species circumscription, biogeography and conservation of coralligenous habitats. J Phycol. 2019;55:473–492. 30657167 10.1111/jpy.12837

[47] Coutinho LM, Penelas Gomes F, Nasri Sissini M, Vieira-Pinto T, Muller de Oliveira Henriques MC, Oliveira MC, et al. Cryptic diversity in non-geniculate coralline algae: a new genus *Roseolithon* (Hapalidiales, Rhodophyta) and seven new species from the Western Atlantic. Eur J Phycol. 2021; doi: 10.1080/09670262.2021.1950839

[48] Hayward BW, Holzmann M, Grenfell HR, Pawlowski J, Triggs CM. Morphological distinction of molecular types in Ammonia—Towards a taxonomic revision of the world’s most commonly misidentified foraminifera. Mar Micropaleontol. 2004;50:237–271.

[49] Pawlowski J, Holzmann M, Tyszka J. New supraordinal classification of Foraminifera: Molecules meet morphology. Mar Micropaleontol. 2013;100:1–10.

[50] Parker JH. Ultrastructure of the test wall in modern porcelaneous foraminifera: implications for the classification of the Miliolida. J Foramin Res. 2017;47:136–174.

[51] Sáez AG, Probert I, Geisen M, Quinn P, Young JR, Medlin LK. Pseudo-cryptic speciation in coccolithophores, P Natl Acad Sci USA. 2003;100:7163–7168. 10.1073/pnas.1132069100 PMC165847 12759476

[52] Cuif J-P, Lecointre G, Perrin C, Tillier A, Tillier S. Patterns of septal biomineralization in Scleractinia compared with their 28S rRNA phylogeny: A dual approach for a new taxonomic framework. Zool Scripta. 2003;32:459–473.

[53] Stolarski J. Three-dimensional micro-and nanostructural characteristics of the scleractinian coral skeleton: A biocalcification proxy. Acta Palaeontol Pol. 2003;48:497–530.

[54] Budd AF, Stolarski J. Searching for new morphological characters in the systematics of scleractinian reef corals: Comparison of septal teeth and granules between Atlantic and Pacific Mussidae. Acta Zool. 2009;90:142–165.

[55] Huang D, Arrigoni R, Benzoni F, Fukami H, Knowlton N, Smith ND, et al. Taxonomic classification of the reef coral family Lobophylliidae (Cnidaria: Anthozoa: Scleractinia). Zool J Linn Soc. 2016;178:436–481.

[56] Basso D. Deep rhodolith distribution in the Pontian Islands, Italy: a model for the paleoecology of a temperate sea. Palaeogeogr Palaeoclimatol Palaeoecol. 1998;137:173–187.

[57] Bressan G, Babbini L. Marine biodiversity of Italian coast: Corallinales of the Mediterranean Sea: guide to the identification. Biol Mar Medit. 2001;10:235–237.

[58] Bosence DWJ. Description and classification of rhodoliths (rhodoids, rhodolites). In: Perit T. editors. Coated Grains. Springer Verlag, 1983. p. 225–42.

[59] Basso D, Fravega P, Vannucci G. Fossil and living corallinaceans related to the Mediterranean endemic species *Lithophyllum racemus* (Lamarck) Foslie. Facies. 1996;35:275–292.

[60] Basso D, Babbini L, Kaleb S, Bracchi VA, Falace A. Monitoring deep Mediterranean rhodolith beds. Aquatic Conserv: Mar Freshw Ecosyst. 2016;26:549–561.

[61] Sañé E, Chiocci FL, Basso D, Martorelli E. Environmental factors controlling the distribution of rhodoliths: an integrated study based on seafloor sampling, ROV and side scan sonar data, offshore the W-Pontine Archipelago. Cont Shelf Res. 2016;129:10–22.

[62] Chimienti G, Rizzo L, Kaleb S, Falace A, Fraschetti S, Giosa FD, et al. Rhodolith Beds Heterogeneity along the Apulian Continental Shelf (Mediterranean Sea). J Mar Sci Eng. 2020;8:813.

[63] Rendina F, Kaleb S, Caragnano A, Ferrigno F, Appolloni L, Donnarumma L, et al. Distribution and characterization of deep rhodolith beds off the Campania coast (SW Italy, Mediterranean Sea). Plants. 2020;9:985. 10.3390/plants9080985 PMC7463569 32759681

[64] Frezza V, Argenti L, Bonifazi A, Chiocci FL, Di Bella L, Ingrassia M, et al. Benthic foraminiferal assemblages and rhodolith facies evolution in Post-LGM sediments from the Pontine Archipelago Shelf (Central Tyrrhenian Sea, Italy). Geosciences. 2021;11:179.

[65] Caragnano A, Rodondi G, Basso D, Peña V, Le Gall L, Rindi F. Circumscription of *Lithophyllum racemus* (Corallinales, Rhodophyta) from the Western Mediterranean Sea reveals the species *Lithophyllum pseudoracemus* sp. nov. Phycologia. 2020;59:584–597.

[66] Basso D, Rodondi G, Mari M. A comparative study between *Lithothamnion minervae* and the type material of *Millepora fasciculata* (Corallinales, Rhodophyta). Phycologia. 2004;43:215–223.

[67] Adey WH, Adey PJ. Studies on the biosystematics and ecology of the epilithic crustose Corallinaceae of the British Isles. Brit Phycol J. 1973;8:343–407.

[68] Johansen HW. Coralline Algae: A First Synthesis. Boca Raton, USA: CRC Press (Taylor & Francis); 1981.

[69] Ballabio D. A MATLAB toolbox for Principal Component Analysis and unsupervised exploration of data structure. Chemometr Intell Lab. 2015;149(Pt B):1–9, doi: 10.1016/j.chemolab.2015.10.003

[70] Piazza G, Valsecchi C, Sottocornola G. Deep learning applied to SEM images for supporting marine coralline algae classification. Diversity. 2021;13:640. doi: 10.3390/ d13120640.

[71] Basso D, Caragnano A, Rodondi G. Trichocytes in *Lithophyllum kotschyanum* and *Lithophyllum* spp. (Corallinales, Rhodophyta) from the NW Indian Ocean. J. Phycol. 2014;50:711–717. 26988454 10.1111/jpy.12197

[72] Cabioch J, Giraud G. Structural aspects of biomineralization in the coralline algae (calcified Rhodophyceae). In: Barry SC, Leadbeater BSC, Riding RE, editors. Biomineralization in lower plants and animals, USA, Oxford University Press, 1986. p. 141–56.

[73] Borowitzka MA, Larkum AWD, Nockolds CE. A scanning electron microscope study of the structure and organization of the calcium carbonate deposits of algae. Phycologia. 1974;13:195–203.

[74] Borowitzka MA. Morphological and cytological aspects of algal calcification. Intl Rev Cytology. 1982;74:127–160.

[75] Nash MC, Adey W. Anatomical structure overrides temperature controls on magnesium uptake-Calcification in the Arctic/subarctic coralline algae *Leptophytum leave* and *Kvaleya epilaeve* (Rhodophyta; Corallinales). Biogeosciences. 2018;15:781–795.

[76] Nash MC, Diaz-Pulido G, Harvey AS, Adey W. Coralline algal calcification: A morphological and process-based understanding. PLoS One. 2019;14:e022 1396. doi: 10.1371/journal.pone.0221396 31557180 PMC6762179

[77] Weiner S, Dove PM. An overview of biomineralization processes and the problem of the vital effect. Rev Mineral Geochem. 2003;54:1–29.

[78] Raz S, Weiner S, Addadi L. Formation of high-magnesian calcites via an amorphous precursor phase: possible biological implications. Adv Mater. 2000;12:38–42.

[79] Mass T, Giuffre AJ, Sun C-Y, Stifler CA, Frazier MJ, Neder M, et al. Amorphous calcium carbonate particles form coral skeletons. P Natl Acad Sci. 2017;201707890. doi: 10.1073/pnas.1707890114 28847944 PMC5604026

[80] De Yoreo JJ, Gilbert PU, Sommerdijk NA, Penn RL, Whitelam S, Joester D, et al. Crystallization by particle attachment in synthetic, biogenic, and geologic environments. Science. 2015;349:6247. 10.1126/science.aaa6760 26228157

[81] Chave KE. Aspects of biochemistry of magnesium.1. Calcareous and marine organisms. J Geol. 1954;62:266–283.

