# Supplementary material for: Calcification traits for cryptic species identification: Insights into coralline biomineralization
Source: PLoS One. 2022 Oct 3;17(10):e0273505. doi: 10.1371/journal.pone.0273505 (PMC9529143; doi:10.1371/journal.pone.0273505)
Supplement: S1 Table — Testing the difference of the perithallial cell area of L. racemus (DB661, DB867), L. pseudoracemus (DB768, DB835), L. cf. racemus DB865, and L. cf. pseudoracemus DB866. Statistically significant p-values are given in bold. Kruskal-Wallis test significance at α = 0.05; Dunn’s test significant at p ≤ α/2. (DOCX) [file pone.0273505.s001.docx]

Table S1: Results of statistical tests performed to evaluate the differences of the perithallial cell area in *L. racemus* (DB661, DB867), *L. pseudoracemus* (DB768, DB835), *L.* cf. *racemus* DB865, and *L.* cf. *pseudoracemus* DB866. Statistically significant p-values are given in bold. Kruskal-Wallis test significance at α = 0.05; Dunn’s test significant at p ≤ α/2.

| **Kruskal-Wallis test**  **(Perithallial cell area)** | | | | | |
| --- | --- | --- | --- | --- | --- |
|  | Df | | χ² | | P |
| SITE | 5 | | 88.104 | | **<2.2e-16** |
| **Dunn's test** | | | | | |
| Comparisons by SITE (Bonferroni) | | | | | |
| Z | DB768 | DB835 | DB661 | DB867 | DB865 |
| P.adjusted |  |  |  |  |  |
| DB835 | 1.609 |  |  |  |  |
|  | 0.807 |  |  |  |  |
| DB661 | 2.121 | 4.250 |  |  |  |
|  | 0.254 | **0.000** |  |  |  |
| DB867 | -5.464 | -7.646 | -3.996 |  |  |
|  | **0.000** | **0.000** | **0.000** |  |  |
| DB865 | -4.321 | -6.290 | -2.758 | 0.971 |  |
|  | **0.000** | **0.000** | 0.044 | 1.000 |  |
| DB866 | -4.076 | -6.055 | -2.467 | 1.284 | 0.291 |
|  | **0.000** | **0.000** | 0.102 | 1.000 | 1.000 |
