# Supplementary material for: Calcification traits for cryptic species identification: Insights into coralline biomineralization
Source: PLoS One. 2022 Oct 3;17(10):e0273505. doi: 10.1371/journal.pone.0273505 (PMC9529143; doi:10.1371/journal.pone.0273505)
Supplement: S2 Table — Testing the differences of the perithallial SC thickness in L. racemus (DB661, DB867), L. pseudoracemus (DB768, DB835), L. cf. racemus DB865, and L. cf. pseudoracemus DB866. Statistically significant p-values are given in bold. ANOVA test significance at α = 0.05; Tukey’s test significant at p ≤ α. (DOCX) [file pone.0273505.s002.docx]

Table S2: Results of statistical tests performed to evaluate the differences of the perithallial SC thickness in *L. racemus* (DB661, DB867), *L. pseudoracemus* (DB768, DB835), *L.* cf. *racemus* DB865, and *L.* cf. *pseudoracemus* DB866. Statistically significant p-values are given in bold. ANOVA test significance at α = 0.05; Tukey’s test significant at p ≤ α.

| **One-way ANOVA test**  **(Perithallial SC thickness)** | | | | | |
| --- | --- | --- | --- | --- | --- |
|  | Df | Sum sq | Mean sq | F value | Pr(>F) |
| SAMPLE | 5 | 0.326 | 0.065 | 9.432 | **0.000** |
| Residuals | 251 | 1.735 | 0.007 |  |  |
| Shapiro-Wilk normality test | | | P=0.838 | | |
| Bartlett's K-squared | | | P=0.103 | | |
| **Tukey test** | | | | | |
| Multiple comparisons of means | | | | | |
| SAMPLE | Mean difference | 95% confidence interval | | P.adjusted | |
|  |  | lower bound | upper bound |  |  |
| DB768-DB661 | -0.070 | -0.116 | -0.023 | **0.000** | |
| DB835-DB661 | -0.031 | -0.076 | 0.015 | 0.383 | |
| DB867-DB661 | 0.033 | -0.022 | 0.088 | 0.509 | |
| DB865-DB661 | 0.021 | -0.039 | 0.081 | 0.916 | |
| DB866-DB661 | -0.053 | -0.106 | 0.000 | 0.054 | |
| DB835-DB768 | 0.039 | -0.005 | 0.083 | 0.110 | |
| DB867-DB768 | 0.103 | 0.049 | 0.156 | **0.000** | |
| DB865-DB768 | 0.091 | 0.032 | 0.150 | **0.000** | |
| DB866-DB768 | 0.017 | -0.035 | 0.069 | 0.933 | |
| DB867-DB835 | 0.064 | 0.011 | 0.116 | **0.008** | |
| DB865-DB835 | 0.052 | -0.007 | 0.110 | 0.115 | |
| DB866-DB835 | -0.022 | -0.073 | 0.029 | 0.816 | |
| DB865-DB867 | -0.012 | -0.078 | 0.054 | 0.995 | |
| DB866-DB867 | -0.086 | -0.145 | -0.026 | **0.001** | |
| DB866-DB865 | -0.074 | -0.138 | -0.009 | **0.015** | |
